# Increased sensitivity of primary aniridia limbal stromal cells to travoprost, leading to elevated migration and MMP-9 protein levels, *in vitro*

**DOI:** 10.1371/journal.pone.0326967

**Published:** 2025-06-26

**Authors:** Shuailin Li, Tanja Stachon, Shanhe Liu, Zhen Li, Shao-Lun Hsu, Swarnali Kundu, Fabian N. Fries, Berthold Seitz, Maryam Amini, Shweta Suiwal, Nóra Szentmáry

**Affiliations:** 1 Dr. Rolf M. Schwiete Center for Limbal Stem Cell and Congenital Aniridia Research, Saarland University, Homburg/Saar, Germany; 2 Department of Ophthalmology, Saarland University Medical Center, Homburg/Saar, Germany; Faculty of Medicine of Tunis, TUNISIA

## Abstract

**Purpose:**

In congenital aniridia, not only limbal epithelial cells but also limbal stromal cells may contribute to the development of aniridia associated keratopathy (AAK). Secondary glaucoma affects 50–75% of patients with congenital aniridia, and prostaglandin analogs are commonly used for conservative treatment. This study aimed to explore the effect of travoprost on corneal limbal stromal cells from healthy (LSCs) and congenital aniridia subjects (AN-LSCs), *in vitro*.

**Materials and methods:**

Cells were extracted from aniridia (AN-LSCs) (n=7) and healthy donors (LSCs) (n=7). In culture, the cells were treated with travoprost at concentrations ranging from 0.039–40 μg/mL for 20 minutes. Cell viability, proliferation and migration were determined to assess the effect of travoprost on AN-LSCs and LSCs. Analysis of inflammation-, retinoic acid signaling-, and apoptosis-related genes and proteins was performed using qPCR, Western blot, and ELISA. One-way ANOVA was used to analyze cell viability and proliferation. The Mann–Whitney test was applied to compare between-group differences, while the Friedman test was used to assess within-group differences.

**Results:**

Both in LSCs and AN-LSCs, travoprost treatment at 0.078 μg/mL and higher concentrations significantly reduced cell viability (p≤0.033; p<0.001) and proliferation decreased both in LSCs and AN-LSCs at 40 μg/mL travoprost concentration (p=0.006; p=0.002). At 6 and 12 hours, 0.313 μg/mL travoprost significantly increased the migration rate of AN-LSCs (p=0.021; p=0.021). AN-LSCs displayed lower *PAX6* and *JNK* (*MAPK8*) mRNA (p<0.001) but higher *MMP-3, MMP-9, ADH7, FABP5* and *VEGFA* mRNA levels (p≤0.037) than LSCs. *PTGFR* and *JNK* mRNA levels, MMP9 and ADH7 protein levels increased significantly in AN-LSCs after 0.313 μg/mL travoprost treatment (p≤0.039), while NF-κB and ADH7 protein levels decreased significantly in LSCs using 0.313 μg/mL travoprost (p=0.039; p<0.001).

**Conclusions:**

Travoprost may affect viability, proliferation, and migration of both LSCs and AN-LSCs, with AN-LSCs exhibiting greater sensitivity than LSCs. Additionally, travoprost may regulate MMP-9 expression in AN-LSCs via the JNK signaling pathway. Furthermore, in AN-LSCs, travoprost treatment does not lead to a decrease in NF-κB and ADH7 protein levels.

## Introduction

Mutation in the paired-box gene 6 (*PAX6*) is the primary causative factor of congenital aniridia (CA) [1–3], which is a rare disease characterized by varying degrees of iris tissue hypoplasia. In addition, congenital aniridia is associated with a spectrum of ocular abnormalities that can affect multiple parts of the eye, including lens anomalies, glaucoma, macular and optic nerve hypoplasia. [1–4].

Patients with congenital aniridia usually develop progressive corneal opacification, called aniridia associated keratopathy (AAK) [5], which typically results in deterioration of visual acuity over time [6]. One of its main causative factors is the progressive limbal stem cell deficiency (LSCD) [7,8]. Additional mechanisms underlying the disease are complex and likely involve multiple factors, such as impaired corneal wound healing due to disrupted extracellular matrix metabolism, defective differentiation of corneal epithelial cells, resulting in increased cellular fragility, and decreased expression of adhesion molecules in individuals with heterozygous *PAX6* mutations, making epithelial cells more vulnerable to mechanical stress [9]. Besides limbal epithelial cells, our recent studies also described the potential role of limbal stromal cells in AAK [10–12]. In addition, PAX6 and keratocyte-specific markers are differentially expressed in aniridia limbal stromal cells compared to healthy controls [12].

Fifty to seventy-five percent of patients with congenital aniridia develop secondary glaucoma [13–16], therefore, either conservative or surgical antiglaucomatous treatment must be initiated during lifetime [7]. Most patients with aniridia associated glaucoma (AAG) are diagnosed during infancy or adolescence [17]. The selection of the antiglaucomatous medication should be carefully considered in patients with AAG, given their AAK and the potential need for future filtering surgery to lower intraocular pressure (IOP) [13]. Prostaglandin analogs (PGAs) are commonly used as conservative treatment of AAG [18] and are commonly considered as the first choice of topical treatment, also in CA patients [13]. Nevertheless, PGAs are known for their proinflammatory effect, which may be disadvantageous for congenital aniridia eyes, with ongoing ocular surface inflammation, as one of the main characteristics of the disease [19–21]. Fries et al. [22] analyzed the potential effect of antiglaucomatous conservative treatment on AAK development/progression. Although a worsening effect of the different antiglaucomatous eyedrops on AAK could not be determined, AAK was significantly worse when using 4 different antiglaucomatous drugs [22]. Nevertheless, the effect of antiglaucomatous medication on AAK needs further evaluation.

Travoprost is a prostaglandin F2α (PGF2α) analog, which was approved by the US FDA in 2001 [23]. At the prostaglandin false positive (FP) receptor site, travoprost has 100% agonism, so it can trigger matrix metalloproteinases (MMPs), which can increase the outflow of the aqueous humor from the uveoscleral pathway by remodeling the extracellular matrix of the ciliary muscle and sclera and widening the connective tissue gap, thereby reducing IOP [24,25]. The effect of travoprost on development and progression of AAK has not been analyzed yet.

Disfunction of the limbal stem cell niche, including limbal epithelial cells and limbal stromal cells is responsible for AAK development. Previous studies described changes in inflammation-, retinoic acid signaling-related genes and proteins, as well as matrix metalloproteinases (MMPs) and mitogen-activated protein kinases (MAPKs) in congenital aniridia limbal epithelial cells and limbal stromal cells [5,26–31]. In addition, an inflammatory environment may also trigger the apoptotic cascade with an effect on cell function [32,33]. Some studies analyzed the effects of travoprost-containing eye drops on healthy human corneal epithelial cells [34–37], and our previous study also analyzed its effect on limbal epithelial cells with PAX6 knockdown, using the siRNA based cell model [38]. Limbal stromal cells have shown the ability to remodel pathological stromal tissue, suppress inflammation, and restore corneal transparency [39]. Nevertheless, to the best of our knowledge, the effect of travoprost on limbal stromal cells has not been reported to date. The purpose of our study was to explore the effect of travoprost on primary human limbal stromal cells from healthy (LSCs) and congenital aniridia subjects (AN-LSCs), *in vitro*.

## Materials and methods

### Ethical approval and consent to participate

The Ethics Committee of Saarland, Germany granted approval for this study (Approval No. 124/23). Our research started on June 22nd, 2023, and ended on November 30th, 2024. Our research was conducted in compliance with the Declaration of Helsinki. Informed consent was obtained from all participants with CA prior to their surgeries.

### Cell culture

Limbal biopsies from congenital aniridia patients were obtained from the Department of Ophthalmology, Saarland University Medical Center, Homburg/Saar, Germany. Control limbal biopsies were collected from corneal donors from the Klaus Faber Center for Corneal Diseases and LIONS Cornea Bank Saar-Lor-Lux, Trier/Westpfalz, in Homburg/Saar, Germany. These tissues were collected from surgical remnants following the use of donor corneas for transplantation. In our experiments, we utilized 1.5 mm limbal biopsies from 7 aniridia eyes (patients aged 39.9 ± 20.0 years, range 13–68 years; 42.9% male) and 7 eyes from healthy donors (aged 50.4 ± 16.1 years, range 29–74 years; 42.9% male). Before surgery, all aniridia patients had slit-lamp examination to assess their AAK Grade, following the criteria set by Lagali et al [40,41]. Descriptive data for the congenital aniridia and healthy samples are summarized in Table 1. Among the seven patients with congenital aniridia, five had a nonsense *PAX6* pathogenic variant, while two had a splicing variant.

**Table 1:** Information on healthy and aniridia subjects.

Healthy Subjects			Aniridia Subjects			
Donor No.	Age (years)	Gender	Donor No.	Age (years)	Gender	AAK Grade
1	29	Male	1	13	Male	4
2	74	Female	2	44	Female	5
3	32	Male	3	50	Female	3
4	56	Female	4	68	Female	3
5	58	Female	5	40	Male	3
6	45	Male	6	50	Male	4
7	59	Female	7	14	Female	4

AN-LSCs and LSCs were isolated following the method outlined by Chai et al. [42]. First, the 1.5 mm biopsy punch was used to extract tissue from the limbal region of the cornea. The tissue was then digested with collagenase A (Hoffmann-La Roche, Basel, Switzerland) at 37°C for 24 hours. Limbal epithelial cells (LECs) and LSCs were separated using CellTrics filters (Sysmax, Norderstedt, Germany), with Phosphate Buffered Saline (PBS) (Merck, Sigma-Aldrich, Taufkirchen, Germany) being used to flush the filter. LSCs were collected from the resulting flowthrough. After centrifugation, the LSCs were stored in liquid nitrogen until further experiments. Prior to measurements, LSCs were cultured in Dulbecco’s modified Eagle’s medium (DMEM/F12) (Thermo Fisher Scientific, Waltham, MA, USA) supplemented with 5% fetal calf serum (FCS) (Thermo Fisher Scientific, Waltham, MA, USA) and 1% penicillin-streptomycin (P/S) (Sigma-Aldrich, St. Louis, MO, USA), and incubated at 37°C with 95% humidity and 5% CO_2_.

### Travoprost treatment

Travoprost (Sigma-Aldrich, St. Louis, MO, USA) was diluted to a concentration of 40 μg/mL (0.004%) in unsupplemented DMEM/F12. This 40 μg/mL solution was further serially diluted by factors of 2–2^10^, yielding travoprost concentrations of 40 μg/mL, 20 μg/mL, 10 μg/mL, 5 μg/mL, 2.5 μg/mL, 1.25 μg/mL, 0.625 μg/mL, 0.313 μg/mL, 0.156 μg/mL, 0.078 μg/mL, and 0.039 μg/mL, respectively.

Cell viability and proliferation assays were conducted in 96-well plates, while the migration assay was performed in 12-well plates. AN-LSCs and LSCs were seeded into either 96-well or 12-well plates in DMEM/F12 supplemented with 5% FCS and 1% P/S. Once cells reached 80–90% confluence, the culture medium was replaced with different concentrations of travoprost containing solutions or with unsupplemented DMEM/F12 as a negative control. After 20 minutes exposure, the solutions were removed, and the cells were rinsed with PBS before adding fresh DMEM/F12 medium to each well.

For RNA and protein isolation, AN-LSCs and LSCs were seeded into 75-cm² culture flasks containing 10 mL of DMEM/F12 supplemented with 5% FCS and 1% P/S. Once the cells reached full confluence, 10 mL of medium containing 0.078 μg/mL, 0.156 μg/mL, or 0.313 μg/mL of travoprost, or without travoprost as a control was added to the flasks. After 20 minutes, the supernatant was collected, and the cells were harvested for further analysis.

### Cell viability assay

Cell viability was assessed using the Cell Proliferation Kit II (XTT) (Roche, Sigma-Aldrich, Mannheim, Germany) following the manufacturer’s instructions. For the 96-well culture plates, a solution was prepared by combining 5 mL of XTT labeling reagent with 0.1 mL of electron coupling reagent. Both reagents were thawed and mixed just before use. A 50 μL aliquot of the mixture was then added to each well, and the plates were incubated at 37°C for 150 minutes. Optical density was measured at 450 nm (with a reference wavelength of 690 nm) using a Tecan Infinite F50 Absorbance Microplate Reader (TECAN Deutschland GmbH, Crailsheim, Germany).

### Cell proliferation assay

Cell proliferation was assessed using the ELISA-BrdU (colorimetric) kit (Roche, Sigma-Aldrich, Mannheim, Germany). Briefly, cells were incubated with 10 μL of BrdU labeling reagent for 3 hours at 37°C. Following this, the BrdU labeling reagent was removed and replaced with 200 μL of FixDenat solution per well for 30 minutes. Afterwards, a mixture of anti-BrdU-POD and antibody dilution solution was added, followed by rinsing the cells three times with 200 μL of PBS. Upon adding the stop solution to the substrate solution, color changes were immediately visible. Optical density values were obtained using the previously mentioned Tecan Infinite F50 Absorbance Microplate Reader.

### Cell migration assay

A scratch assay was used to assess cell migration. Initially, three parallel reference lines, spaced 5 mm apart, were drawn at the bottom of each well of the 12-well cell culture plates. AN-LSCs and LSCs were then seeded into the 12-well plates with 1.5 mL of DMEM/F12. Once the cells reached 80% confluence, a scratch was made in each well perpendicular to the reference lines using 100 μL pipette tips (Eppendorf AG, Hamburg, Germany). Treatment was applied as previously described using medium containing 0.078 μg/mL, 0.156 μg/mL, or 0.313 μg/mL of travoprost, or without travoprost as a control. Phase-contrast images were captured immediately after treatment (0 hours) and then at 6, 12, and 24-hour intervals. In each well, four distinct scratch regions along the reference lines were photographed for further analysis. The images were processed using ImageJ (https://imagej.nih.gov/ij/), and the Migration Rate (MR) was calculated using the formula: MR = [Wound area (0 h) – Wound area (6/12/24 h)]/ Wound area (0 h).

### RNA isolation and cDNA synthesis

RNA was extracted using the Total RNA Purification Plus Micro Kit (Norgen Biotek, Ontario, Canada), following the manufacturer’s instructions. Briefly, genomic DNA was first removed using the Genomic DNA Removal Column. Following three wash steps with the RNA Purification Micro Column to eliminate impurities, RNA was eluted and centrifuged to obtain purified total RNA. The isolated RNA was stored at −80°C until it was used for cDNA synthesis, which was carried out using the One Taq® RT-PCR Kit (New England Biolabs INC, Frankfurt, Germany). For each sample, 500 ng of total RNA served as the template for cDNA synthesis, according to the manufacturer’s instructions. The resulting cDNA was then stored at −20°C for future use.

### Quantitative PCR

Table 2 lists the primers used for quantitative Polymerase Chain Reaction (qPCR). The reaction mixture for qPCR had a total volume of 9 µL, consisting of 1 µL of the specific primer solution, 5 µL of SYBR Green Mix (Vazyme, Nanjing, China), and 3 µL of nuclease-free water. According to the manufacturer’s protocol, 1 µL of cDNA was added to the mixture. The qPCR was performed using a QuantStudio 5 real-time PCR system (Thermo Fisher Scientific, Waltham, MA, USA) under the following conditions: an initial denaturation at 95°C for 10 s, followed by 60°C for 30 s, and a final step at 95°C for 15 s, repeated for 40 cycles. All samples were measured in duplicate, with normalization done using Tata-binding protein (TBP) and Beta-glucuronidase (GUSB) as endogenous reference genes, following the ΔΔCT method. Fold changes (2^ΔΔCT^) were computed for statistical analysis, with untreated LSCs serving as the reference group (fold change = 1).

**Table 2:** qPCR primers information.

Primer	Gene Symbol	Catalog number	Manufacturer
ADH7	ADH7	QT00000217	Qiagen N.V., Venlo, Netherlands
ALDH1A1	ALDH1A1	QT00013286	Qiagen N.V., Venlo, Netherlands
Bax	BAX	QT00031192	Qiagen N.V., Venlo, Netherlands
Bcl-2	BCL2	QT00025011	Qiagen N.V., Venlo, Netherlands
caspase3	CASP3	QT00023947	Qiagen N.V., Venlo, Netherlands
CRABP-2	CRABP2	QT00063434	Qiagen N.V., Venlo, Netherlands
FABP5	FABP5	QT00225561	Qiagen N.V., Venlo, Netherlands
GUSB	GUSB	QT00046046	Qiagen N.V., Venlo, Netherlands
IL-6	IL6	QT00083720	Qiagen N.V., Venlo, Netherlands
IL-8	CXCL8	QT00000322	Qiagen N.V., Venlo, Netherlands
MAPK1	MAPK1	QT00065933	Qiagen N.V., Venlo, Netherlands
MAPK3	MAPK3	QT02589314	Qiagen N.V., Venlo, Netherlands
MAPK8	MAPK8	QT00091056	Qiagen N.V., Venlo, Netherlands
MAPK14	MAPK14	QT00079345	Qiagen N.V., Venlo, Netherlands
MMP-2	MMP2	QT02395778	Qiagen N.V., Venlo, Netherlands
MMP-3	MMP3	QT00060025	Qiagen N.V., Venlo, Netherlands
MMP-9	MMP9	QT00040040	Qiagen N.V., Venlo, Netherlands
NF-κB	RELA	QT02324308	Qiagen N.V., Venlo, Netherlands
PAX6	PAX6	QT00071169	Qiagen N.V., Venlo, Netherlands
PPARγ	PPARG	QT00029841	Qiagen N.V., Venlo, Netherlands
PTGES2	PTGES2	QT00082068	Qiagen N.V., Venlo, Netherlands
PTGFR	PTGFR	QT02449846	Qiagen N.V., Venlo, Netherlands
TBP	TBP	QT00000721	Qiagen N.V., Venlo, Netherlands
TNF-α	TNF	QT00029162	Qiagen N.V., Venlo, Netherlands
VEGFA	VEGFA	QT01010184	Qiagen N.V., Venlo, Netherlands

### Protein quantification

After cells in a 75-cm² flask reached confluence, they were lysed in 40 µL of RIPA buffer (Thermo Fisher Scientific, Waltham, MA, USA). Protein concentrations were determined using the Pierce™ BCA Protein Assay Kit (Thermo Fisher Scientific, Waltham, MA, USA). The absorbance measurements were performed on the Tecan Infinite F50 Absorbance Microplate Reader at a wavelength of 560 nm. Bovine serum albumin was used as the standard, and all measurements were carried out in duplicates.

### Western blot analysis

For Western blot analysis, 20 μg of total protein was heated in 5 µL of Laemmli buffer at 95°C for 5 minutes and then applied to a 4–12% NuPage™ Bis-Tris SDS precast gel (Invitrogen, Waltham, MA, USA). For molecular weight estimation, 2.5 μL of Precision Plus Protein™ All Blue Prestained Protein Standards (Bio-Rad Laboratories, Hercules, USA) was loaded into the first well. NuPAGE™ MOPS SDS Running Buffer (20×) (Thermo Fisher Scientific, Waltham, MA, USA) was used as the electrophoresis buffer. Following protein separation, transfer to a nitrocellulose membrane was carried out using the Trans-Blot Turbo Transfer System (Bio-Rad Laboratories, Hercules, USA), following the device’s preset protocol for transferring high molecular weight proteins via semi-dry blotting.

The membrane was treated with No-Stain™ Protein labeling reagent (ThermoFisher Scientific™ GmbH, Dreieich, Germany) to quantify total protein in each lane for normalization. It was then washed three times, each with 10 mL of Western Froxx washing solution (BioFroxx GmbH, Einhausen, Germany) for 5 minutes. Afterwards, the membrane was incubated overnight at 4°C with the primary antibodies, listed in Table 3. The primary antibodies were prepared using a combined blocking and secondary antibody solution (WesternFroxx anti-Rabbit/Mouse HRP; BioFroxx GmbH, Einhausen, Germany). Following the incubation, the membrane was washed three times with 10 mL of washing solution to remove the antibody solution.

**Table 3:** Western blot antibody information.

Antibody	Dilution	Catalog number	Manufacturer
ADH7 Polyclonal Antibody	1:1000	PA5–98484	Thermo Fisher Scientific, Waltham, USA
Aldehyde dehydrogenase 1-A1/ALDH1A1 Antibody (H-4)	1:100	sc-374076	Santa Cruz Biotechnology, Dallas, USA
Caspase3/p17/p19 Polyclonal antibody	1:1000	19677-1-AP	Proteintech Group, Rosemont, USA
CRABP2 Monoclonal antibody	1:2500	66468-1-lg	Proteintech Group, Rosemont, USA
FABP5 Polyclonal antibody	1:1000	12348-1-AP	Proteintech Group, Rosemont, USA
JNK Polyclonal antibody	1:5000	24164-1-AP	Proteintech Group, Rosemont, USA
NF-κB p65 (D14E12) XP Rabbit mAb	1:1000	8242	Cell Signaling Technology, Danvers, USA
PAX6 Rabbit Polyclonal antibody	1:500	12323-1-AP	Proteintech Group, Rosemont, USA
PPAR Gamma Polyclonal antibody	1:1000	16643-1-AP	Proteintech Group, Rosemont, USA
PTGES2 Polyclonal antibody	1:500	10881-1-AP	Proteintech Group, Rosemont, USA

Protein bands were visualized using the Western Lightning Plus Chemiluminescence Reagent (PerkinElmer Inc., Waltham, MA, USA), with chemiluminescence detected by the iBright™ CL1500 Imaging System (Thermo Fisher Scientific, Waltham, MA, USA). After detection, the membrane was stripped using Western Froxx stripping solution (BioFroxx GmbH, Einhausen, Germany) to allow for additional antibody incubations.

### Enzyme linked immunosorbent assay (ELISA)

The supernatant levels of IL-6, IL-8, MMP-2, MMP-3, MMP-9, and VEGFA were quantified using DuoSet® ELISA Kits (R&D Systems, Bio-Techne, Minneapolis, USA), listed in Table 4, following the manufacturer’s protocol. All assays were conducted in duplicate with the Tecan Infinite F50 Absorbance Microplate Reader at 450 nm. Data were normalized against total protein content to determine concentrations.

**Table 4:** ELISA kit information.

ELISA kit	Catalog number	Manufacturer
Human IL-6 DuoSet ELISA	DY206	R&D Systems, Bio-Techne, Minneapolis, USA
Human IL-8/CXCL8 DuoSet ELISA	DY208	R&D Systems, Bio-Techne, Minneapolis, USA
Human MMP-2 DuoSet ELISA	DY902	R&D Systems, Bio-Techne, Minneapolis, USA
Human Total MMP-3 DuoSet ELISA	DY513	R&D Systems, Bio-Techne, Minneapolis, USA
Human MMP-9 DuoSet ELISA	DY911	R&D Systems, Bio-Techne, Minneapolis, USA
Human VEGF DuoSet ELISA	DY293B	R&D Systems, Bio-Techne, Minneapolis, USA

### Statistical analysis

Statistical analyses and diagram preparation were performed using GraphPad Prism version 9.2.0. Data from cell viability and proliferation assays are presented as mean ± standard deviation (SD), while migration assay, qPCR, Western blot, and ELISA results are shown as median with interquartile range (IQR).

Normality was assessed using the Shapiro–Wilk test. For normally distributed data, one-way ANOVA was used to analyze cell viability and proliferation. The Mann–Whitney test was applied to assess between-group differences (LSCs vs. AN-LSCs), while the Friedman test was used for within-group comparisons (i.e., the same cells treated with different concentrations of travoprost) regarding migration assay results, mRNA expression (2^∆∆Ct^ values), and protein levels in LSCs and AN-LSCs. P values < 0.05 were considered statistically significant.

A priori power analysis was conducted using G*Power (version 3.1) for the Friedman test (repeated measures, within-subjects design). Assuming a moderate effect size (Kendall’s W = 0.4), an alpha level of 0.05, and four related conditions, a sample size of 7 independent biological replicates (donors) provides approximately 80% power to detect statistically significant differences.

## Results

### Cell viability and proliferation

**Fig 1:**
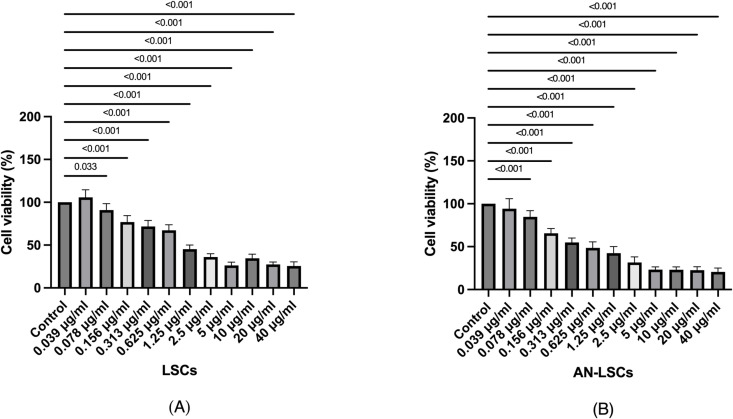
Cell viability of limbal stromal cells (LSCs) and aniridia limbal stromal cells (AN-LSCs) after 20-minute treatment with travoprost at concentrations ranging from 0.039 μg/mL to 40 μg/mL (n = 7). One-way ANOVA was performed, statistically significant p-values (p < 0.05) are indicated in the diagrams. **(A)** Treatment with travoprost at concentrations of 0.156 μg/mL and above led to a significant decrease in LSC viability (p ≤ 0.033). **(B)** Treatment with travoprost at concentrations of 0.156 μg/mL and above resulted in a significant reduction in AN-LSC viability (p < 0.001 for all).

Results of the XTT assay are summarized at Fig 1. Both in LSCs and AN-LSCs, travoprost treatment at 0.078 μg/ml and higher concentrations significantly reduced cell viability, compared to untreated controls (p ≤ 0.033; p < 0.001).

**Fig 2:**
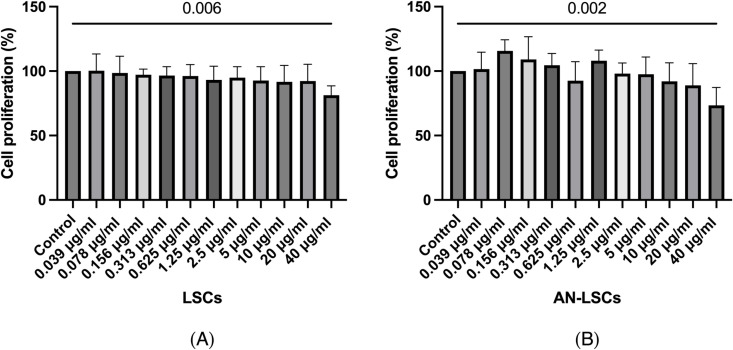
Cell proliferation of limbal stromal cells (LSCs) and aniridia limbal stromal cells (AN-LSCs) after 20-minute treatment with travoprost at concentrations ranging from 0.039 μg/mL to 40 μg/mL, using the ELISA-BrdU (colorimetric) kit (n = 7). One-way ANOVA was performed, statistically significant p-values (p < 0.05) are indicated in the diagrams. **(A)** LSC proliferation significantly decreased after treatment with 40 μg/mL travoprost (p = 0.006). **(B)** AN-LSC proliferation significantly decreased after treatment with 40 μg/mL travoprost (p = 0.002).

Results of the BrdU assay are displayed at Fig 2. Proliferation decreased both in LSCs and AN-LSCs at 40 μg/mL travoprost concentration, compared to untreated controls (p = 0.006; p = 0.002).

### Cell migration

**Fig 3:**
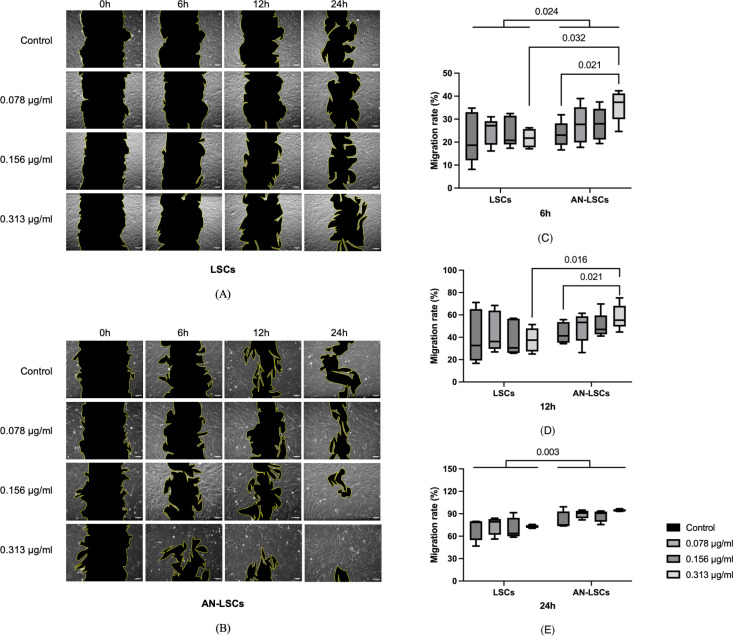
Images of the scratch assays (A, B) and migration rates (C, D, E) for limbal stromal cells (LSCs) and aniridia limbal stromal cells (AN-LSCs) at 0, 6, 12, and 24 hours following a 20-minute treatment with varying concentrations of travoprost (0.078 μg/mL, 0.156 μg/mL, and 0.313 μg/mL) (n = 5). Scale bar: 50 μm. The Mann–Whitney test was applied to compare between-group differences (LSCs vs. AN-LSCs), while the Friedman test was used to assess within-group differences (i.e., the same cells treated with varying concentrations of travoprost). Migration rate values are presented as median with interquartile range (IQR). Significant p values (< 0.05) are highlighted at the diagrams. At, 6 and 24 hours, the migration rate in AN-LSCs was significantly higher, than in LSCs (p = 0.024; p = 0.003) (C, E). At 6 h, AN-LSCs treated with 0.313 μg/mL travoprost had a significantly higher migration rate than untreated AN-LSCs (p = 0.021) and AN-LSCs treated with 0.313 μg/mL travoprost exhibited a higher migration rate than LSCs treated with the same concentration (p = 0.032) (C). At 12 h, AN-LSCs treated with 0.313 μg/mL travoprost had a significantly higher migration rate than untreated AN-LSCs (p = 0.021) and AN-LSCs treated with 0.313 μg/mL travoprost exhibited a higher migration rate than LSCs treated with the same concentration (p = 0.016) (D).

Images and results of the scratch assay are shown at Fig 3. From 0 to 24 hours both in the LSCs and AN-LSCs groups, the scratched area decreased gradually (Fig 3A, B).

At 6 and 24 hours, the migration rate of AN-LSCs was significantly higher than that of LSCs (p = 0.024; p = 0.003). At 6 hours, AN-LSCs treated with 0.313 μg/mL travoprost exhibited a significantly higher migration rate compared to untreated AN-LSCs (p = 0.021). AN-LSCs treated with 0.313 μg/mL travoprost showed a significantly higher migration rate than LSCs treated with the same concentration (p = 0.032). At 12 hours, AN-LSCs treated with 0.313 μg/mL travoprost exhibited a significantly higher migration rate compared to untreated AN-LSCs (p = 0.021). AN-LSCs treated with 0.313 μg/mL travoprost showed a significantly higher migration rate than LSCs treated with the same concentration (p = 0.016). By 24 hours, treatment with different concentrations of travoprost did not significantly increase migration rate in either cell type compared to their respective untreated controls (p ≥ 0.618) (Fig 3C–E).

### PAX6 mRNA and protein levels

**Fig 4:**
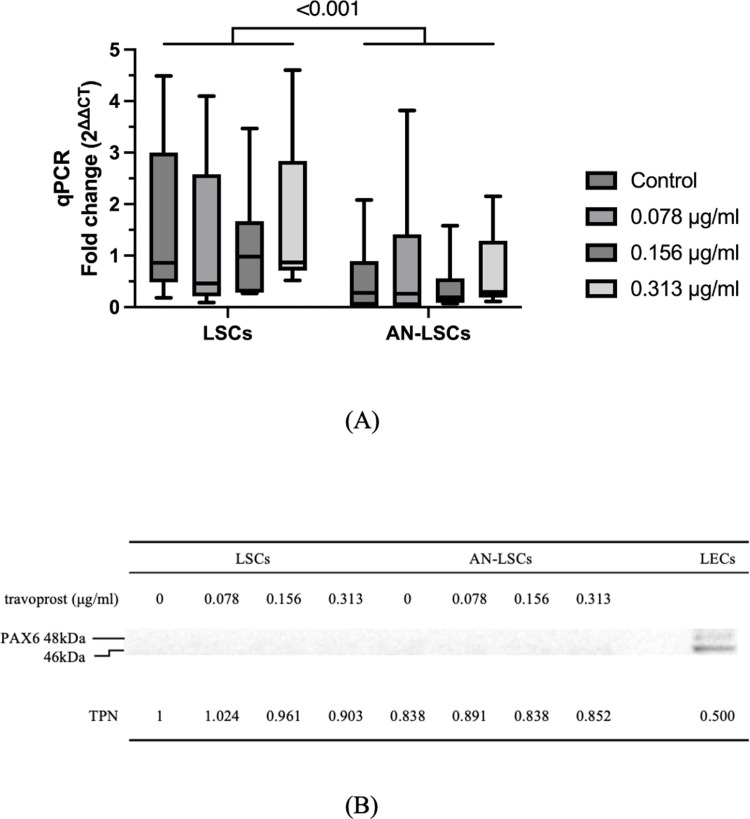
*PAX6* mRNA levels and a representative PAX6 western blot from limbal stromal cells (LSCs) and aniridia limbal stromal cells (AN-LSCs) following a 20-minute treatment with varying concentrations of travoprost (0.078 μg/mL, 0.156 μg/mL, and 0.313 μg/mL) (n = 7). The Mann–Whitney test was applied to compare between-group differences (LSCs vs. AN-LSCs), while the Friedman test was used to assess within-group differences (i.e., the same cells treated with varying concentrations of travoprost). mRNA values are displayed on a logarithmic scale (log₂) and presented as median with interquartile range (IQR). Significant p values (< 0.05) are highlighted at the diagrams. *PAX6* mRNA levels were significantly lower in AN-LSCs than in LSCs (p < 0.001), nevertheless, a significant effect of travoprost treatment within LSC and AN-LSC groups on PAX6 mRNA expression levels could not be observed (p ≥ 0.115) (A). PAX6 protein level was too low to be detected in LSCs and AN-LSCs, while its expression could be observed in our positive controls, limbal epithelial cells (LECs) (B).

*PAX6* mRNA levels were significantly lower in AN-LSCs compared to LSCs (p < 0.001). However, no significant effect of travoprost treatment on *PAX6* mRNA expression was observed within either the LSC or AN-LSC groups (p ≥ 0.115). PAX6 protein levels were undetectable in both LSCs and AN-LSCs, whereas its expression was observed in the positive control limbal epithelial cells (LECs) (Fig 4).

### Inflammation-related genes

**Fig 5:**
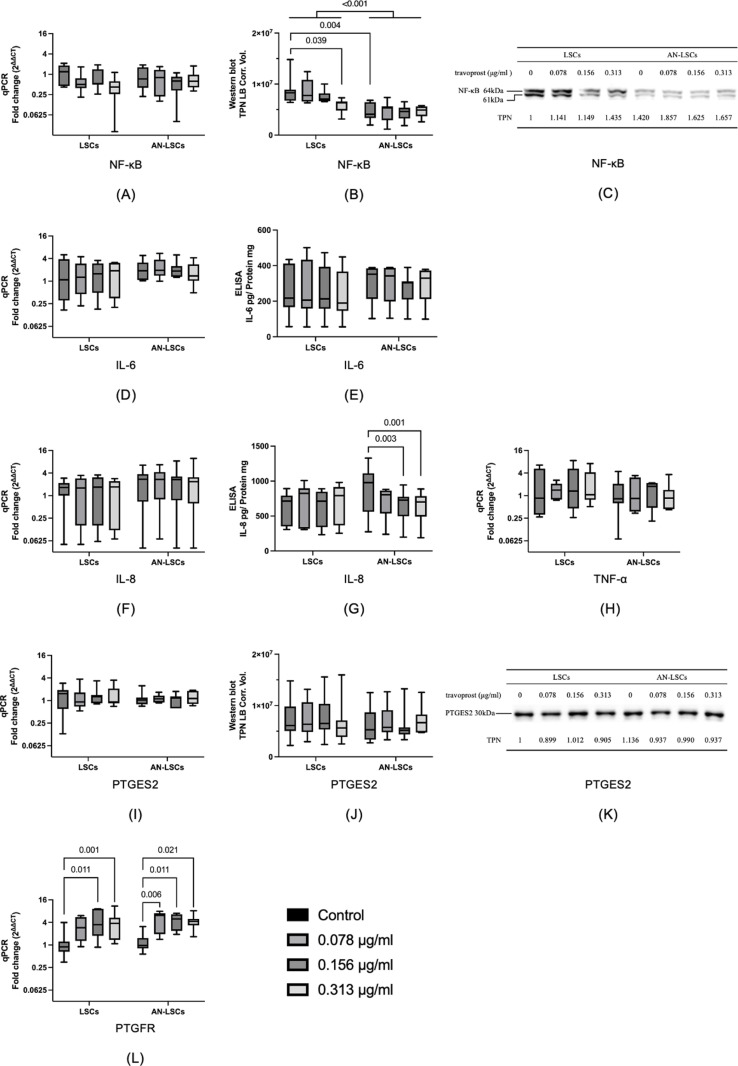
Inflammation related markers *NF-κB, IL-6, IL-8, TNF-α*, *PTGES2*, and *PTGFR* mRNA and NF-κB, IL-6, IL-8, and PTGES2 protein levels in limbal stromal cells (LSCs) and aniridia limbal stromal cells (AN-LSCs) following a 20-minute treatment with varying concentrations of travoprost (0.078 μg/mL, 0.156 μg/mL, and 0.313 μg/mL) (n = 7) (A-L). NF-κB and PTGES2 protein levels have been determined using western blot, IL-6 and IL-8 protein levels using ELISA. The Mann–Whitney test was applied to compare between-group differences (LSCs vs. AN-LSCs), while the Friedman test was used to assess within-group differences (i.e., the same cells treated with varying concentrations of travoprost). mRNA values are shown on a logarithmic scale (log₂) and presented as median with interquartile range (IQR); protein values are also expressed as median with IQR. Significant p values (< 0.05) are highlighted at the diagrams. TPN LB Corr. Vol. means total lane protein local background corrected volume at the Y-axis. *NF-κB*, *IL-6*, *IL-8*, *TNF-α*, *PTGES2* and *PTGFR* mRNA levels and IL-6 and PTGES2 protein levels did not differ significantly between LSCs and AN-LSCs groups or between any subgroups (p ≥ 0.078) **(A-L)**. Nevertheless, NF-κB protein levels were significantly lower (p < 0.001) **(B, C)** in AN-LSCs, than in LSCs. Baseline NF-κB protein levels were also significantly lower (p = 0.004) **(B, C)** in AN-LSCs, than in LSCs. In addition, in LSCs, 0.313 μg/mL travoprost treatment significantly reduced NF-κB protein levels, compared to untreated LSCs (p = 0.039) **(B, C)**. In AN-LSCs, 0.156 and 0.313 μg/mL travoprost treatment significantly reduced IL-8 protein levels, compared to untreated AN-LSCs (p = 0.003; p = 0.001) **(G)**. Treatment with 0.156 and 0.313 μg/mL travoprost significantly increased *PTGFR* mRNA levels in LSCs (p ≤ 0.011), while treatment with 0.078, 0.156, and 0.313 μg/mL travoprost significantly elevated *PTGFR* mRNA levels in AN-LSCs, compared to their respective untreated controls (p ≤ 0.021) **(L)**.

*NF-κB, IL-6, IL-8, TNF-α*, *PTGES2* and *PTGFR* mRNA levels, as well as IL-6, and PTGES2 protein levels, did not differ significantly between LSCs and AN-LSCs or among any subgroups (p ≥ 0.078). However, NF-κB protein levels were significantly lower in AN-LSCs than in LSCs (p < 0.001). Furthermore, baseline untreated NF-κB protein levels were also significantly lower in AN-LSCs than in LSCs (p = 0.004). In addition, in LSCs, 0.313 μg/mL travoprost treatment significantly reduced NF-κB protein levels, compared to untreated LSCs (p = 0.039). In AN-LSCs, 0.156 and 0.313 μg/mL travoprost treatment significantly reduced IL-8 protein levels, compared to untreated AN-LSCs (p = 0.003; p = 0.001). Furthermore, treatment with 0.156 and 0.313 μg/mL of travoprost significantly improved *PTGFR* mRNA levels (p ≤ 0.011) in LSCs, and treatment with 0.078, 0.156, and 0.313 μg/mL of travoprost significantly improved *PTGFR* mRNA levels (p ≤ 0.021) in AN-LSCs, compared to their respective untreated controls (Fig 5).

### Mitogen-activated protein kinases (MAPKs)

**Fig 6:**
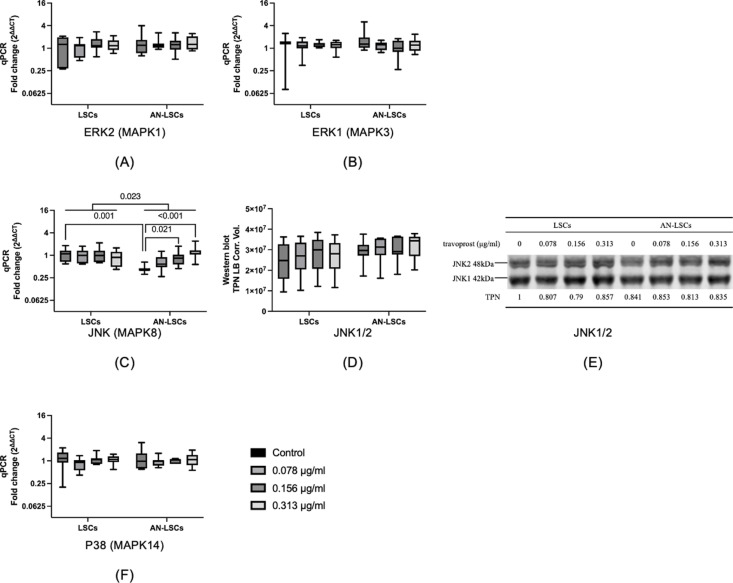
Mitogen-activated protein kinases (MAPKs) *ERK2 (MAPK1), ERK1 (MAPK3), JNK (MAPK8)*, and *p38 (MAPK14)* mRNA levels and JNK1/2 protein levels in limbal stromal cells (LSCs) and aniridia limbal stromal cells (AN-LSCs) following a 20-minute treatment with varying concentrations of travoprost (0.078 μg/mL, 0.156 μg/mL, and 0.313 μg/mL) (n = 7) (A-F). JNK1/2 protein level has been determined using western blot. The Mann–Whitney test was applied to compare between-group differences (LSCs vs. AN-LSCs), while the Friedman test was used to assess within-group differences (i.e., the same cells treated with varying concentrations of travoprost). mRNA values are shown on a logarithmic scale (log₂) and presented as median with interquartile range (IQR); protein values are also expressed as median with IQR. Significant p values (< 0.05) are highlighted at the diagrams. TPN LB Corr. Vol. means total lane protein local background corrected volume at the Y-axis. *ERK2 (MAPK1), ERK1 (MAPK3)* and *p38 (MAPK14)* mRNA levels and JNK1/2 protein levels did not differ significantly between LSCs and AN-LSCs (p ≥ 0.073) **(A-F)**. Nevertheless, *JNK* mRNA level was significantly lower in untreated AN-LSCs, than in LSCs (p = 0.001). In addition, 0.156 and 0.313 μg/mL travoprost treatment significantly increased *JNK* mRNA levels in AN-LSCs, compared to untreated AN-LSCs (p = 0.021; p < 0.001) **(C)**^‌‌^.

*ERK2 (MAPK1), ERK1 (MAPK3)*, and *p38 (MAPK14)* mRNA levels, as well as JNK1/2 protein levels, did not differ significantly between LSCs and AN-LSCs (p ≥ 0.073). However, *JNK* mRNA levels were significantly lower in untreated AN-LSCs compared to LSCs (p = 0.001). Moreover, treatment with 0.156 and 0.313 μg/mL travoprost significantly increased *JNK* mRNA levels in AN-LSCs compared to untreated AN-LSCs (p = 0.021; p < 0.001) (Fig 6).

### Matrix metalloproteinases (MMPs)

**Fig 7:**
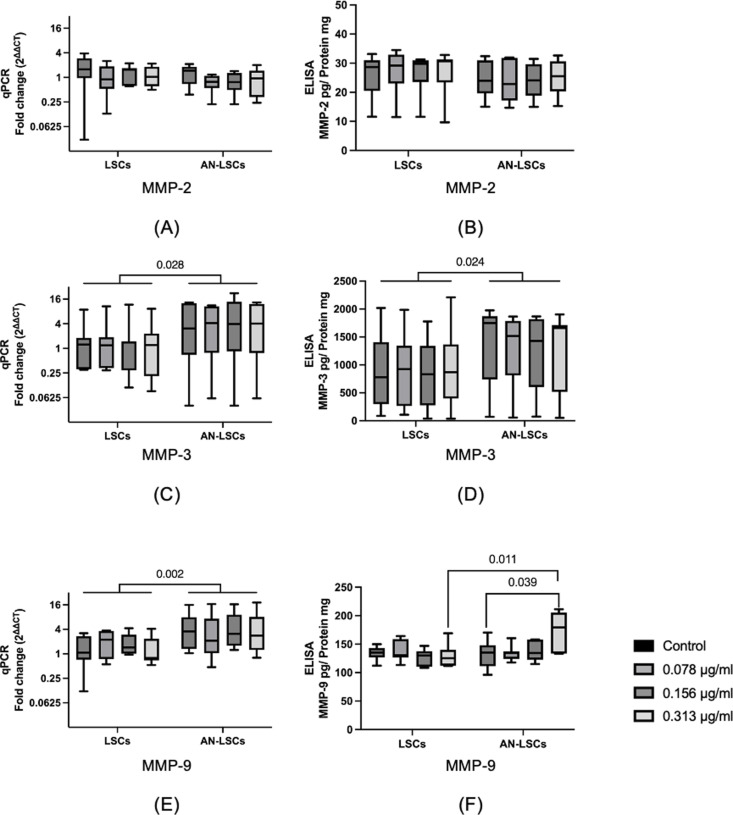
Matrix metalloproteinases MMP-2, MMP-3, and MMP-9 mRNA and protein levels in limbal stromal cells (LSCs) and aniridia limbal stromal cells (AN-LSCs) following a 20-minute treatment with varying concentrations of travoprost (0.078 μg/mL, 0.156 μg/mL, and 0.313 μg/mL) (n = 7) (A-F). MMP-2, MMP-3, and MMP-9 protein levels have been determined using ELISA. The Mann–Whitney test was applied to compare between-group differences (LSCs vs. AN-LSCs), while the Friedman test was used to assess within-group differences (i.e., the same cells treated with varying concentrations of travoprost). mRNA values are shown on a logarithmic scale (log₂) and presented as median with interquartile range (IQR); protein values are also expressed as median with IQR. Significant p values (< 0.05) are highlighted at the diagrams. *MMP-3* and *MMP-9* mRNA levels and MMP-3 protein levels were significantly higher in AN-LSCs than in LSCs groups (p ≤ 0.028) **(C, D, E)**. In addition, MMP-9 protein level was significantly higher in 0.313 μg/mL travoprost treated AN-LSCs, than in LSCs (p = 0.011) and MMP-9 protein level was significantly higher in 0.313 μg/mL travoprost treated AN-LSCs, than in untreated AN-LSCs (p = 0.039) **(F)**. There were no further significant differences between any other analysed groups (p ≥ 0.068).

*MMP-3* and *MMP-9* mRNA levels, as well as MMP-3 protein levels, were significantly higher in AN-LSCs compared to LSCs (p ≤ 0.028). Additionally, MMP-9 protein levels were significantly higher in AN-LSCs treated with 0.313 μg/mL travoprost compared to LSCs (p = 0.011). Furthermore, MMP-9 protein levels were also significantly elevated in 0.313 μg/mL travoprost-treated AN-LSCs compared to untreated AN-LSCs (p = 0.039). No other significant differences were observed among the analyzed groups (p ≥ 0.068). (Fig 7).

### Apoptosis-related genes

**Fig 8:**
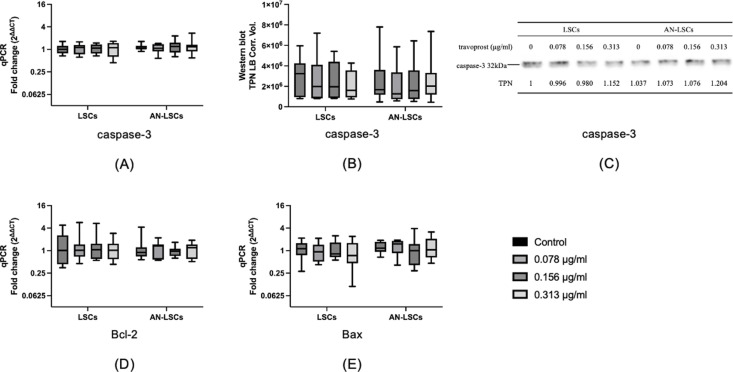
Apoptotic markers *caspase-3, Bcl-2*, and *Bax* mRNA and caspase-3 protein levels in limbal stromal cells (LSCs) and aniridia limbal stromal cells (AN-LSCs) following a 20-minute treatment with varying concentrations of travoprost (0.078 μg/mL, 0.156 μg/mL, and 0.313 μg/mL) (n = 7) (A-E). Caspase-3 protein level has been determined using western blot. The Mann–Whitney test was applied to compare between-group differences (LSCs vs. AN-LSCs), while the Friedman test was used to assess within-group differences (i.e., the same cells treated with varying concentrations of travoprost). mRNA values are shown on a logarithmic scale (log₂) and presented as median with interquartile range (IQR); protein values are also expressed as median with IQR. Significant p values (< 0.05) are highlighted at the diagrams. TPN LB Corr. Vol. means total lane protein local background corrected volume at the Y-axis.

Regarding apoptosis-related genes, no significant differences were observed in any of the analyzed mRNA or protein levels between LSCs and AN-LSCs or among any of the subgroups (p ≥ 0.277) (Fig 8).

There was no significant difference in any of the analysed mRNA and protein levels between LSCs and AN-LSCs or between any of the subgroups (p ≥ 0.277).

### Retinoic acid signaling pathway-related genes

**Fig 9:**
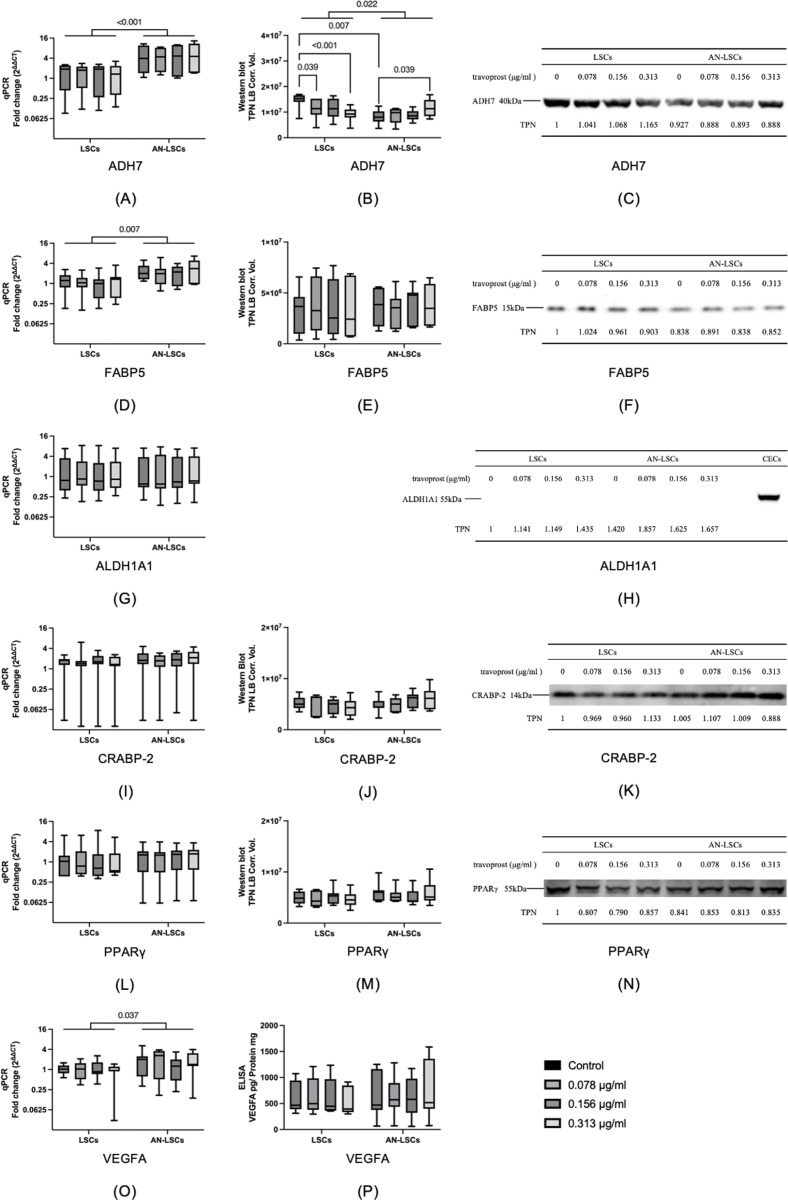
*ADH7, FABP5, ALDH1A1, CRABP-2, PPARγ* and *VEGFA* mRNA and ADH7, FABP5, CRABP-2, PPARγ and VEGFA protein levels in limbal stromal cells (LSCs) and aniridia limbal stromal cells (AN-LSCs) following a 20-minute treatment with varying concentrations of travoprost (0.078 μg/mL, 0.156 μg/mL, and 0.313 μg/mL) (n = 7) (A-P). ADH7, FABP5, CRABP-2 and PPARγ protein levels have been determined using western blot and VEGFA protein levels using ELISA. The Mann–Whitney test was applied to compare between-group differences (LSCs vs. AN-LSCs), while the Friedman test was used to assess within-group differences (i.e., the same cells treated with varying concentrations of travoprost). mRNA values are shown on a logarithmic scale (log₂) and presented as median with interquartile range (IQR); protein values are also expressed as median with IQR. Significant p values (< 0.05) are highlighted at the diagrams. TPN LB Corr. Vol. means total lane protein local background corrected volume at the Y-axis. *ADH7, FABP5* and *VEGFA* mRNA levels were significantly higher in AN-LSCs, than in LSCs (p ≤ 0.037) while ADH7 protein level was significantly lower in AN-LSCs, than in LSCs (p = 0.022) **(A, B, D, O)**. In addition, ADH7 protein level was significantly lower in untreated AN-LSCs, than in LSCs (p = 0.007). Furthermore, 0.078 and 0.313 μg/mL travoprost treatment significantly decreased ADH7 protein levels in LSCs (p = 0.039; p < 0.001) and 0.313 μg/mL travoprost treatment significantly increased ADH7 protein levels in AN-LSCs, compared to their untreated controls (p = 0.039) **(B, C)**. ALDH1A1 protein level was too low to be detected in LSCs and AN-LSCs, while its expression could be observed in our positive controls, corneal epithelial cells (CECs) **(H)**. There was no further significant difference in any of the analysed mRNA and protein levels between LSCs and AN-LSCs or between any of the subgroups (p ≥ 0.065).

*ADH7, FABP5*, and *VEGFA* mRNA levels were significantly higher in AN-LSCs compared to LSCs (p ≤ 0.037), while ADH7 protein levels were significantly lower in AN-LSCs, than in LSCs (p = 0.022). Additionally, ADH7 protein levels were significantly lower in untreated AN-LSCs than in LSCs (p = 0.007). Furthermore, 0.078 and 0.313 μg/mL travoprost treatment significantly decreased ADH7 protein levels in LSCs (p = 0.039; p < 0.001) and 0.313 μg/mL travoprost treatment significantly increased ADH7 protein levels in AN-LSCs (p = 0.039). However, no further significant differences were observed in any of the analyzed mRNA or protein levels between LSCs and AN-LSCs or among any subgroups (p ≥ 0.065) (Fig 9).

## Discussion

This study investigates the effects of different travoprost concentrations on LSCs and AN-LSCs, examining cell viability, proliferation, migration, and the expression of genes and proteins related to inflammation, retinoic acid signaling, apoptosis, as well as MMPs and MAPKs. While several previous studies have investigated the effects of travoprost on normal immortalized corneal epithelial cells [37,43–45], to our knowledge, this is the first study to explore the impact of antiglaucomatous drugs on primary limbal stromal cells and on primary limbal stromal cells from patients with CA. Our findings indicate that, at specific concentrations, travoprost significantly influences the viability, proliferation, and migration of AN-LSCs and regulates the expression of some related genes and proteins.

In our study, we selected 40 μg/mL travoprost concentration as the upper limit, based on the concentration of the commercially available Travatan Z formulation (0.004% = 40 μg/mL), which reflects the maximum initial exposure on the ocular surface following topical instillation. As there are currently no published data on the actual concentration of travoprost reaching the limbal region in humans, and considering that *in vivo* concentrations decrease due to tear dilution and clearance, we applied a 1:2 serial dilution, reducing the concentration from 40 μg/mL to 0.039 μg/mL. This approach was intended to cover a broad potential range of travoprost exposure at the limbus.

Travoprost is an isopropyl ester prodrug that is hydrolyzed by esterases into a biologically active free acid form. This free acid is structurally similar to fluprostenol and other PGF2α analogs [46]. Therefore, travoprost is a synthetic ester prodrug of a PGF2α analog and functions as a selective agonist of the PTGFR [47]. *PTGFR* mRNA levels increased significantly in both LSCs and AN-LSCs following various travoprost treatments, compared to untreated controls, showing that travoprost acts through *PTGFR* to influence the expression of downstream genes and proteins.

The XTT assay results revealed that exposure to 0.156 μg/mL or higher concentrations of travoprost for 20 minutes led to a significant decrease in LSCs and AN-LSCs viability. Additionally, the BrdU assay results showed that a 20-minute treatment with travoprost at 40 μg/mL concentration significantly decreased AN-LSCs and LSCs proliferation. Both the decrease in viability and proliferation following travoprost treatment showed greater significance in AN-LSCs, with lower p-values compared to LSCs. LSCs possess mesenchymal characteristics, playing a role in supporting angiogenesis and differentiating into various corneal cell types [48,49]. Studies have also shown that PAX6 can induce the proliferation and differentiation of bone marrow mesenchymal stem cells [50]. The lower *PAX6* mRNA level observed in AN-LSCs compared to LSCs may explain their increased sensitivity to travoprost-induced reductions in proliferation.

The scratch assay results showed that travoprost did not significantly affect the migration ability of LSCs. However, in AN-LSCs, treatment with 0.313 μg/mL travoprost for 20 minutes significantly enhanced cell migration. Further analysis revealed that at 6 and 24 hours, AN-LSCs consistently exhibited a higher migration rate than LSCs, with travoprost treatment further promoting their migration rate. Interestingly, the expression pattern of MMP-9 closely correlated with the migration rate of AN-LSCs, as its mRNA levels were significantly higher than those in LSCs. Additionally, ELISA results demonstrated that a 20-minute treatment with 0.313 μg/mL travoprost significantly increased MMP-9 protein levels in AN-LSCs. Studies suggest that increased MMP-9 expression may enhance the migratory and invasive capabilities of various tumor cell types [51–53]. The mechanism by which prostaglandin analogs lower intraocular pressure in glaucoma patients involves the regulation of MMP levels and extracellular matrix remodeling [54,55]. In our study, travoprost specifically increased MMP-9 expression in AN-LSCs, while MMP-2 and MMP-3 levels remained unchanged. These findings suggest that travoprost may promote AN-LSC migration by upregulating MMP-9. The role of MMP-9 in AAK is not yet fully understood. However, it has been demonstrated that MMP-9 induces extracellular matrix remodeling and enhances cell migration [56]. We hypothesize that the overexpression of MMP-9 may reduce the stability of the corneal stroma by disrupting the orderly arrangement of stromal fibers, thereby affecting corneal transparency [57]. Moreover, the role of MMPs in regulating pathological retinal neovascularization has also been reported [58,59], suggesting that MMP-9 overexpression may also exacerbate corneal neovascularization, which is a key pathological feature in the progression of AAK [5]. However, further studies are needed to elucidate the exact underlying mechanisms.

*PAX6* mRNA expression was significantly lower in AN-LSCs compared to LSCs; however, travoprost treatment did not influence *PAX6* mRNA levels. Analysis of inflammation-related genes revealed that IL-8 protein levels decreased in AN-LSCs following treatment with 0.156 and 0.313 μg/mL travoprost. Western blot analysis showed that baseline NF-κB protein levels were lower in AN-LSCs than in LSCs and travoprost reduced NF-κB protein expression in LSCs, but this effect was absent in AN-LSCs. A study by Tong et al. [44] found that corneal epithelial cells exposed to 40 μg/mL travoprost containing the preservative polyquaternium-1 (PQ-1) for 5 minutes exhibited significantly increased IL-6 and IL-8 expression. Similarly, Paimela et al. [43] reported that corneal epithelial cells treated with artificial tears containing PQ-1 at the same concentration showed significantly elevated IL-6, IL-8, and NF-κB expression. Nevertheless, our study used preservative-free travoprost at lower concentrations, raising the question of whether the increased IL-6 and IL-8 expression in previous studies was due to travoprost itself or the presence of PQ-1, or could this be observed due to the different cell type (epithelial cells vs LSCs). Notably, a study using 40 μg/mL travoprost in mice found no significant increase in IL-6 expression [60].

Regarding the MAPK/ERK and JNK signaling pathways, we found that untreated AN-LSCs exhibited significantly lower *JNK* mRNA levels compared to LSCs. In addition, after travoprost treatment, *JNK* mRNA levels showed an increasing trend. Despite this, Western blot results did not reveal any significant changes in JNK protein expression. Similarly, an *in vivo* mouse study reported no significant increase in pJNK expression following travoprost treatment [60]. The JNK signaling pathway plays a crucial role in various cellular processes, including migration and proliferation [61]. Studies have demonstrated a correlation between JNK expression and the migration rate of various tumor and neuronal cells [62–64]. Additionally, the JNK pathway is known to regulate MMP expression [65,66]. Based on these findings, we hypothesize that travoprost may influence MMP-9 expression via the JNK signaling pathway, thereby modulating AN-LSC migration. Nevertheless, further studies should explore the exact pathways regulating MMP-9 levels, in limbal stromal cells.

Caspase-3 and Bax are pro-apoptotic factors, while Bcl-2 is an anti-apoptotic factor [67]. Our results showed no significant changes in apoptosis-related markers caspase-3, Bax, and Bcl-2 at either the mRNA or protein level, suggesting that travoprost has a limited effect on apoptosis in both LSCs and AN-LSCs. *In vitro* studies [68,69] examining whether travoprost induces apoptosis found that treating conjunctival epithelial cells and fibroblasts with 40 μg/mL travoprost for at least 30 minutes did not increase apoptosis rates. Similarly, an *in vivo* study in mice reported no increase in corneal cell apoptosis following travoprost treatment [60]. These findings align with the results of our present study. However, another *in vitro* study [43] on corneal epithelial cells showed that after exposure to 40 μg/mL travoprost containing the preservative PQ-1 for 5 minutes and 15 minutes, caspase-3 levels increased. Nevertheless, after 30 minutes of exposure, the change in caspase-3 levels was not statistically significant anymore. It remains unclear whether the observed changes in caspase-3 expression were due to travoprost itself or the preservative used in the study.

Notably, travoprost treatment reduced ADH7 protein level in LSCs but increased its level in AN-LSCs. Similarly, our previous study [10] found that ritanserin treatment upregulated ADH7 and FABP5 expression in LSCs. Further studies, with ADH protein substitution in cell culture may potentially elucidate the molecular mechanisms behind these observations. Nevertheless, our findings suggest that travoprost has a limited impact on alterations in the RA signaling pathway in AN-LSCs.

In conclusion, this study highlights the effects of a 20-minute treatment with different concentrations of travoprost on cell viability, proliferation, migration, and gene/protein expression in LSCs and AN-LSCs. However, certain limitations should be acknowledged, including the need for *in vivo* studies and further investigation of varying treatment durations and concentrations. Additionally, we note that variations in *PAX6* pathogenic variants among patients may influence LSC gene expression profiles. Consequently, studies with larger sample sizes are warranted to further validate and strengthen our findings. Future research should explore higher concentrations and extended exposure times to gain a more comprehensive understanding of travoprost’s mechanisms of action.

## Conclusions

Our study results suggest that travoprost influences the viability, proliferation, and migration of LSCs and AN-LSCs, with AN-LSCs exhibiting greater sensitivity to travoprost than LSCs. Additionally, travoprost may regulate MMP-9 expression in AN-LSCs through the JNK signaling pathway, thereby affecting cell function. These findings offer valuable insights for selecting anti-glaucoma medications for patients with congenital aniridia and secondary glaucoma.

## Supporting information

S1 File Ethics approval in German. (PDF)

S2 File Ethics approval in English. (PDF)

S3 File Western blot raw images. (PDF)

S4 File Original Data. (PDF)
