# Supplementary material for: Increased sensitivity of primary aniridia limbal stromal cells to travoprost, leading to elevated migration and MMP-9 protein levels, in vitro
Source: PLoS One. 2025 Jun 26;20(6):e0326967. doi: 10.1371/journal.pone.0326967 (PMC12200743; doi:10.1371/journal.pone.0326967)
Supplement: S1 File — (PDF) [file pone.0326967.s001.pdf]

Ärztekammer
des Saarlandes

Körperschaft
des öffentlichen Rechts

Ärztekammer des Saarlandes · Postfach 10 02 62 · 66002 Saarbrücken
Ethikkommission

Ethikkommission
Geschäftsstelle

Faktoreistraße 4
66111 Saarbrücken

Sachbearbeitung:
D. Bunzel • Telefon (06 81) 40 03 - 218
N. Halder • Telefon (06 81) 40 03 - 216

Telefax (06 81) 40 03 - 43 94
E-Mail: ethikkommission@aeksaar.de
Internet: www.aerztekammer-saarland.de

Frau Prof. Dr. med Nóra Szentmáry
Dr. Rolf M. Schwiete Zentrum für
Limbusstammzellforschung und kongenitale Aniridie
Universitätsklinikum des Saarlandes
Kirrberger Str. 100, Gebäude 22
66424 Homburg/Saar

Unser Zeichen:	Ihr Schreiben vom:	Ihr Zeichen:	Datum:
124/23			21. JUNI 2023

Effekt von antiglaukomatösen Augentropfen auf Apoptose-, Stammzell-und Differenzierungsmarker in humanen primären limbalen Epithelzellen und Fibroblasten von Aniridie-Patienten
Unsere Kenn-Nr. 124/23 (Bitte in jedem Schriftwechsel angeben!)

Sehr geehrte Frau Professorin Szentmáry,

wir nehmen Bezug auf das Schreiben per E-Mail vom 14.06.2023 von Frau Hannah Rapp, mit dem uns die o.g. Studie zur Beurteilung vorgelegt wird.

Die Unterlagen wurden entsprechend § 8 unserer Geschäftsordnung vom Vorsitzenden geprüft, es ist keine Beratung durch die gesamte Kommission erfolgt.

Gegen die Durchführung der o. g. Studie im Prüfzentrum – Dr. Rolf M. Schwiete Zentrum für Limbusstammzellforschung und kongenitale Aniridie, Universitätsklinikum des Saarlandes, Kirrberger Str. 100, Gebäude 22, 66424 Homburg/Saar – bestehen weder berufsrechtliche noch ethische Bedenken.

Wir machen darauf aufmerksam, dass die Ethik-Kommission mit ihrer Stellungnahme lediglich eine Hilfestellung bei der Beurteilung ethischer und rechtlicher Gesichtspunkte eines geplanten Forschungsvorhabens gibt. Verantwortlich für die Planung und Durchführung bleibt der zuständige ärztliche Leiter des Forschungsvorhabens.

Bei Änderungen des Forschungsvorhabens vor oder während der Durchführung bedarf es nochmals eines entsprechenden Antrages vor der Änderung. Bei Änderungen sollten sowohl die Antrags-Nummer als auch die geänderten Passagen in den betreffenden Unterlagen deutlich gekennzeichnet sein, da andernfalls keine zügige Bearbeitung möglich ist. Auch müssen neu teilnehmende Prüfzentren der Ethik-Kommission gemeldet werden.

Die Ethik-Kommission bei der Ärztekammer des Saarlandes ist unter Beachtung der internationalen Richtlinien der ICH, GCP sowie des AMG und der MDR tätig, nach Landesrecht (Saarländisches Heilberufekammergesetz, § 5 Abs. 1) anerkannt und beim Bundesinstitut für Arzneimittel und Medizinprodukte (§ 41a AMG) sowie beim Bundesamt für Strahlenschutz nach § 36 Abs. 1 Strahlenschutzgesetz registriert.

Datenschutzrechtliche Aspekte von Forschungsvorhaben werden durch die Ethikkommission grundsätzlich nur kursorisch geprüft. Dieses Votum / diese Bewertung ersetzt mithin nicht die Konsultation des zuständigen betrieblichen oder behördlichen Datenschutzbeauftragten.

Commerzbank Saarbrücken
IBAN: DE35 5904 0000 0538 9200 00
BIC: COBADEFFXXX

apoBank Saarbrücken
IBAN: DE69 3006 0601 0001 9262 09
BIC: DAAEDEDDXXX

Bank 1 Saar Saarbrücken
IBAN: DE83 5919 0000 0001 5750 07
BIC: SABADE5S

Wir machen ausdrücklich darauf aufmerksam, dass bei erkennbarer Trendwende mit negativem Ergebnis das Forschungsvorhaben abzubrechen ist.

Wir wünschen für die vorgesehene Aufgabe viel Erfolg und wären zur gegebenen Zeit für die Übersendung eines Abschlussberichtes dankbar.

Mit freundlichen Grüßen

Grundmann

Prof. Dr. U. Grundmann
Vorsitzender

Der Bewertung liegen die nachfolgend aufgeführten Unterlagen zugrunde:

- Aufklärung_AN-Glaukom_2023.docx
- Einwilligung_AN-Glaukom_2023.docx
- EK-0002 Elektronische Checkliste_AN-Glaukom_2023.doc
- E-Mail Anschreiben vom 14.06.2023
