# Supplementary material for: Increased sensitivity of primary aniridia limbal stromal cells to travoprost, leading to elevated migration and MMP-9 protein levels, in vitro
Source: PLoS One. 2025 Jun 26;20(6):e0326967. doi: 10.1371/journal.pone.0326967 (PMC12200743; doi:10.1371/journal.pone.0326967)
Supplement: S3 File — (PDF) [file pone.0326967.s003.pdf]

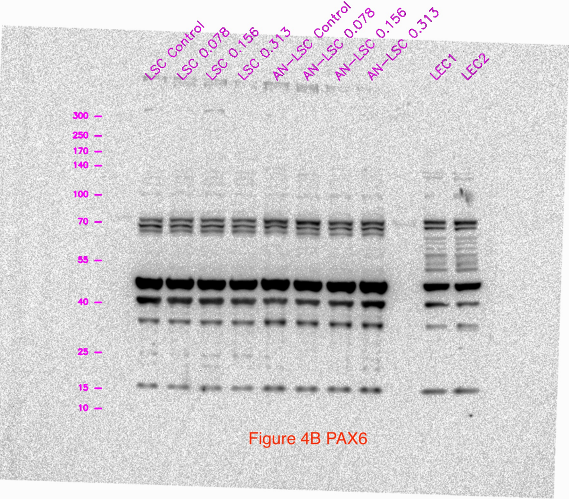

Figure 4B PAX6

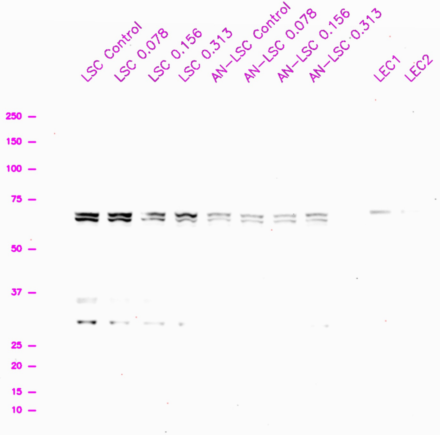

Figure 5C NF-κB

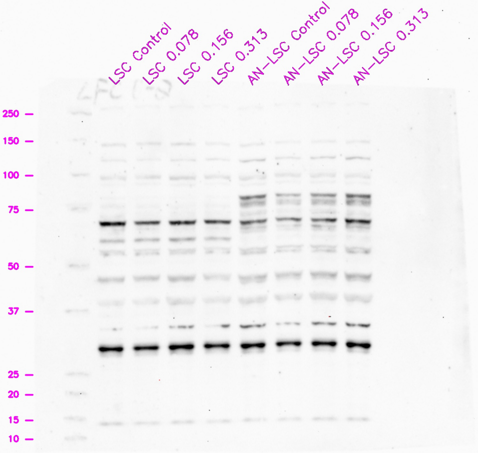

Figure 5K PTGES2

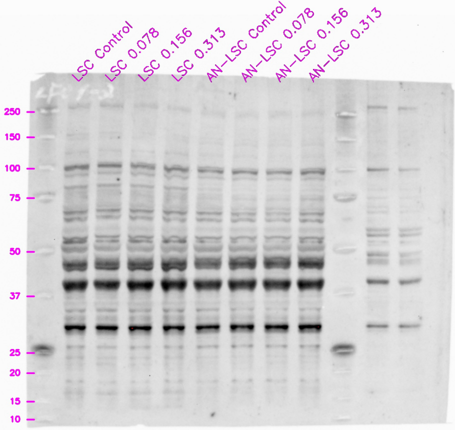

Figure 6E JNK1/2

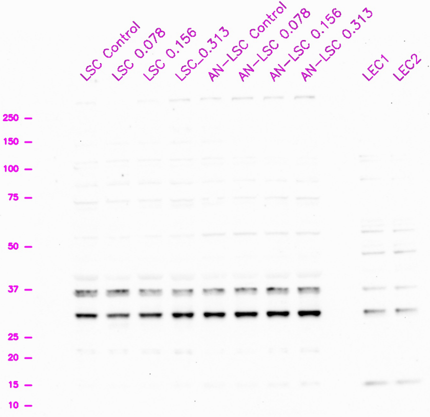

Figure 8C caspase-3

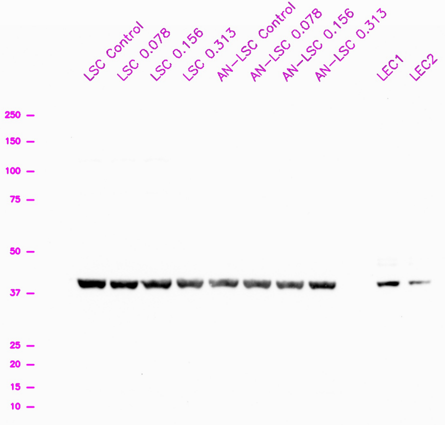

Figure 9C ADH7

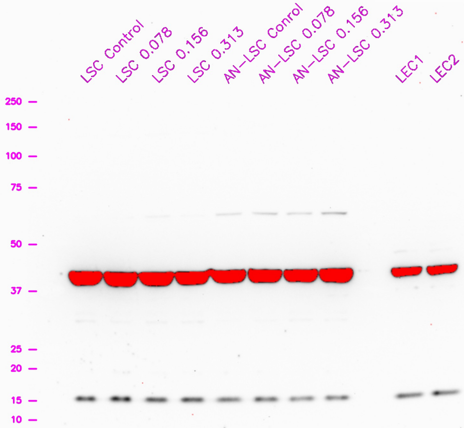

Figure 9F FABP5

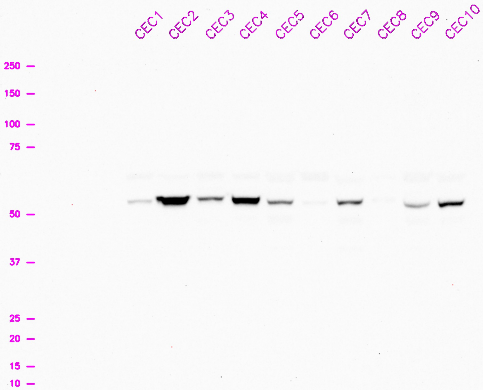

Figure 9H ALDH1A1 positive control

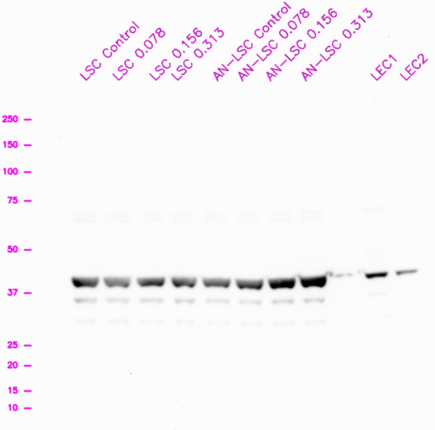

Figure 9H ALDH1A1

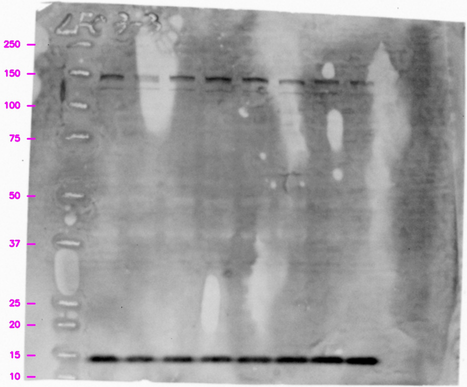

Figure 9K CRABP2

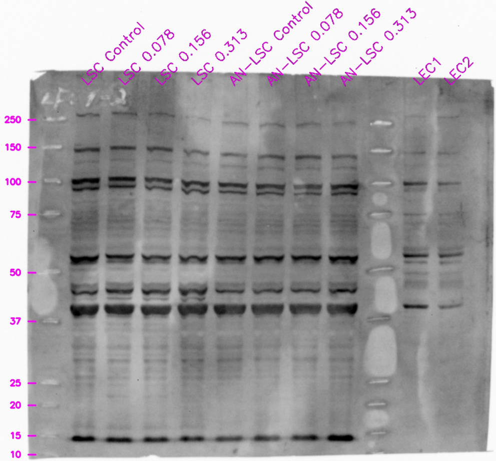

Figure 9N PPARγ
