# Supplementary material for: Increased sensitivity of primary aniridia limbal stromal cells to travoprost, leading to elevated migration and MMP-9 protein levels, in vitro
Source: PLoS One. 2025 Jun 26;20(6):e0326967. doi: 10.1371/journal.pone.0326967 (PMC12200743; doi:10.1371/journal.pone.0326967)
Supplement: S4 File — (PDF) [file pone.0326967.s004.pdf]

Original Data

1. Cell viability

LSCs

Travoprost	Control	0.039μg/mL	0.078μg/mL	0.156μg/mL	0.313μg/mL	0.625μg/mL	1.25μg/mL	2.5μg/mL	5μg/mL	10μg/mL	20μg/mL	40μg/mL
Donor 1	100	106.67	84.83	76.87	73.42	67.43	48.9	37.17	27.72	34.52	26.34	26.28
Donor 2	100	105.74	84.48	67.05	61.13	59.35	40.46	31.78	22.4	28.31	22.24	20.13
Donor 3	100	117.65	86.91	68.9	66.58	62.32	38.73	31	21.32	31.03	26.16	17.61
Donor 4	100	95.58	104.2	84.83	80.43	77.98	51.13	38.05	31.93	38.95	30.34	28.23
Donor 5	100	99.88	97.85	87.31	80.27	73.66	49.31	38.57	29.33	42.37	29.45	27.43
Donor 6	100	116.92	92.11	75.82	71.45	63.04	43.07	33.77	27.08	32.14	30.2	27.76
Donor 7	100	97.7	86.09	76.36	69.14	66.51	45.44	41.86	24.5	34.78	26.73	31.27
Mean	100	105.7	90.92	76.73	71.77	67.18	45.29	36.03	26.33	34.59	27.35	25.53
SD	0	8.85	7.569	7.456	7.037	6.598	4.737	3.964	3.802	4.791	2.898	4.852

AN-LSCs

Travoprost	Control	0.039μg/mL	0.078μg/mL	0.156μg/mL	0.313μg/mL	0.625μg/mL	1.25μg/mL	2.5μg/mL	5μg/mL	10μg/mL	20μg/mL	40μg/mL
Donor 1	100	101.19	87.92	71.05	62.88	59.14	57.06	44.66	28.94	28.85	30.07	29.99
Donor 2	100	79.52	79.57	65	54.26	45.83	40.05	29.87	20.05	20.26	21.08	18.69
Donor 3	100	91.09	88.72	72.23	60.27	58.36	47.88	33.68	25.4	25.31	25.84	23.02
Donor 4	100	91.88	78.26	69.27	51.62	46.53	39.94	27.74	23.19	21.94	19.87	18.59
Donor 5	100	82.55	95.9	64.24	52.47	44.37	34.86	23.4	20.49	19.73	19.07	17.14
Donor 6	100	113.53	87.52	57.96	55.03	42.39	38.73	30.11	21.7	20.86	21.8	17.99
Donor 7	100	99.76	75.53	59.8	48.25	43.29	38.9	31.36	23.04	24.43	20.58	18.4
Mean	100	94.22	84.77	65.65	54.97	48.56	42.49	31.55	23.26	23.05	22.62	20.55
SD	0	11.69	7.208	5.497	5.063	7.106	7.517	6.612	3.09	3.304	3.942	4.57

2. Cell proliferation

LSCs

Travoprost	Control	0.039μg/mL	0.078μg/mL	0.156μg/mL	0.313μg/mL	0.625μg/mL	1.25μg/mL	2.5μg/mL	5μg/mL	10μg/mL	20μg/mL	40μg/mL
Donor 1	100	93.38	93.44	95.56	91.45	85.36	105.61	97.92	89.04	84.18	85.59	78.62
Donor 2	100	104.52	98.09	100.77	100.06	96.56	101.16	103.61	92.6	91.16	100.75	79.23
Donor 3	100	86.05	88.02	94.14	103.11	102.15	104.74	100.76	105.3	100.82	90.4	77.06
Donor 4	100	97.37	93.34	92.94	90.73	96.3	87.27	101.92	106.95	87.46	86.13	79.3
Donor 5	100	103.93	124.83	100.79	105.01	109.2	85.29	87.68	83.38	89.64	97.1	89.07
Donor 6	100	125.59	104.41	103.28	98.69	99.37	89.75	92.23	93.93	114.32	113.37	93.27
Donor 7	100	91.13	87.49	92.55	86.68	84.01	78.84	80.3	77.98	74.24	72.42	72.56
Mean	100	100.3	98.52	97.15	96.53	96.14	93.24	94.92	92.74	91.69	92.25	81.3
SD	0	13	12.98	4.367	6.938	8.95	10.54	8.573	10.65	12.77	13.04	7.228

AN-LSCs

Travoprost	Control	0.039μg/mL	0.078μg/mL	0.156μg/mL	0.313μg/mL	0.625μg/mL	1.25μg/mL	2.5μg/mL	5μg/mL	10μg/mL	20μg/mL	40μg/mL
Donor 1	100	124.96	113.73	76.38	94.71	91.81	92.5	80.48	70.37	61.6	54.79	54.21
Donor 2	100	99.28	109.6	105.32	115.26	79.24	116.15	97.89	102.38	100.59	98.58	74.93
Donor 3	100	111.69	115.26	115.24	98.81	122.29	110.45	103.89	99.58	91.44	78.77	56.66
Donor 4	100	98.68	115.03	100.77	100.57	92.86	102.74	98.74	93.17	96.9	94.26	76.85
Donor 5	100	85.69	116.77	122.62	109.55	90.77	115.93	97.92	98.91	91.44	100.9	90.83
Donor 6	100	90.58	106.25	111.29	96.16	77.07	106.95	102.61	106.84	95.72	99.88	73.41
Donor 7	100	100.31	133.26	131.52	116.77	93.05	111.44	104.65	111.64	106.67	95.48	87.09
Mean	100	101.6	115.7	109	104.5	92.44	108	98.03	97.56	92.05	88.95	73.43
SD	0	13.15	8.569	17.71	9.175	14.75	8.328	8.243	13.37	14.44	16.81	13.87

3. Cell migration

6h

	LSCs				AN-LSCs			
Travoprost	Control	0.078µg/mL	0.156µg/mL	0.313µg/mL	Control	0.078µg/mL	0.156µg/mL	0.313µg/mL
Donor 1	8.15	27.3	20.78	21.73	24.51	31.4	31.54	35.31
Donor 2	34.81	31	32.43	26.22	20.91	17.71	22.79	37.34
Donor 3	31.35	27.13	30.64	25.2	16.62	38.98	27.97	40.05
Donor 4	15.97	21.54	20.6	18.39	23.08	22.01	19.39	24.64
Donor 5	18.66	16.13	17.36	17.09	31.89	27.72	37.47	42.36
25% Percentile	12.06	18.84	18.98	17.74	18.77	19.86	21.09	29.98
Median	18.66	27.13	20.78	21.73	23.08	27.72	27.97	37.34
75% Percentile	33.08	29.15	31.54	25.71	28.2	35.19	34.51	41.21

12h

	LSCs				AN-LSCs			
Travoprost	Control	0.078µg/mL	0.156µg/mL	0.313µg/mL	Control	0.078µg/mL	0.156µg/mL	0.313µg/mL
Donor 1	16.69	36.22	30.55	37.49	41.28	61.43	47.08	54.51
Donor 2	71.15	59.37	56.27	51.41	35.87	26.28	41.12	44.7
Donor 3	59.26	68.45	56.94	44.45	34.3	53.26	49.27	61
Donor 4	21.78	32.3	26.22	25.06	51.56	47.71	44.14	55.33
Donor 5	32.66	26.89	25.73	29.58	55.77	56.19	69.78	75.23
25% Percentile	19.24	29.6	25.98	27.32	35.09	37	42.63	49.61
Median	32.66	36.22	30.55	37.49	41.28	53.26	47.08	55.33
75% Percentile	65.21	63.91	56.61	47.93	53.67	58.81	59.53	68.12

24h

	LSCs				AN-LSCs			
Travoprost	Control	0.078µg/mL	0.156µg/mL	0.313µg/mL	Control	0.078µg/mL	0.156µg/mL	0.313µg/mL
Donor 1	46.81	56.23	58.67	70.6	73.72	81.79	93.69	92.84
Donor 2	79.87	84.12	91.45	71.45	99.28	94.96	88.68	93.17
Donor 3	76.48	77.32	62.51	75.63	75.82	89.79	75.6	96.72
Donor 4	81.64	81.06	64.95	73.79	73.28	91.89	92.68	95.06
25% Percentile	54.23	61.5	59.63	70.81	73.39	83.79	78.87	92.92
Median	78.18	79.19	63.73	72.62	74.77	90.84	90.68	94.12
75% Percentile	81.2	83.36	84.83	75.17	93.42	94.19	93.44	96.31

4. PAX6

PAX6 qPCR

	LSCs				AN-LSCs			
Travoprost	Control	0.078µg/mL	0.156µg/mL	0.313µg/mL	Control	0.078µg/mL	0.156µg/mL	0.313µg/mL
Donor 1	0.48	0.67	0.37	0.91	0.46	0.45	0.17	0.24
Donor 2	0.18	0.09	0.27	0.84				
Donor 3	3	2.58	1.67	2.84	0.5	0.02	0.07	0.11
Donor 4	4.49	4.1	3.47	4.6	2.08	3.82	1.58	2.15
Donor 5	0.86	0.21	1.4	0.52	0.03	0.07	0.22	0.35
Donor 6	1.19	0.46	0.98	0.87	0.01	0.02	0.21	0.21
Donor 7	0.82	0.39	0.28	0.71	0.09	0.61	0.09	1
25% Percentile	0.48	0.21	0.28	0.71	0.025	0.02	0.085	0.185
Median	0.86	0.46	0.98	0.87	0.275	0.26	0.19	0.295
75% Percentile	3	2.58	1.67	2.84	0.895	1.413	0.56	1.288

5. Inflammation related genes

NF-κB qPCR

	LSCs				AN-LSCs			
Travoprost	Control	0.078μg/mL	0.156μg/mL	0.313μg/mL	Control	0.078μg/mL	0.156μg/mL	0.313μg/mL
Donor 1	1.15	0.39	0.52	0.3	0.53	0.61	0.49	0.59
Donor 2	2.11	0.5	0.26	0.26	1.88	0.8	1.1	0.97
Donor 3	0.42	0.52	0.51	0.42	0.22	0.16	0.63	0.62
Donor 4	1.72	1.65	1.89	0.02	1.61	0.92	0.7	0.89
Donor 5	0.46	0.76	0.82	0.62	0.4	0.22	0.33	0.41
Donor 6	1.24	0.21	1.4	1.14	0.73	1.35	0.85	0.32
Donor 7					1.4	1.69	0.04	1.77
25% Percentile	0.45	0.345	0.4475	0.2	0.4	0.22	0.33	0.41
Median	1.195	0.51	0.67	0.36	0.73	0.8	0.63	0.62
75% Percentile	1.818	0.9825	1.523	0.75	1.61	1.35	0.85	0.97

NF-κB Western blot

	LSCs				AN-LSCs			
Travoprost	Control	0.078μg/mL	0.156μg/mL	0.313μg/mL	Control	0.078μg/mL	0.156μg/mL	0.313μg/mL
Donor 1	14812643	12439404.9	7499892.08	6361997.91	4091628.87	2942668.28	3258475.08	3701500.91
Donor 2	8920686	10832443.8	10004973.8	6599517.6	6460572.82	7374253.38	6549884.66	4920763.51
Donor 3	8164922	6337503.01	6567430.61	6463929.69	3350673.1	3994657.97	4776414.5	5626839.7
Donor 4	8288873	6608080.45	6674365.58	4827479.86	3491557.74	5428868.42	4645667.82	5783824.97
Donor 5	6413319	7774164.06	8185544.22	7322594.68	6824480.91	5363690.24	4243763.72	4795288.73
Donor 6	8578136	6589062.16	6958190.2	3160917.12	1983264.6	1181862.91	1866129.92	2601406.87
Donor 7	6765967	9429340.78	7080633.8	5052882.23	5837329.51	5648266.55	5377487.99	5139851.42
25% Percentile	6765967	6589062	6674366	4827480	3350673	2942668	3258475	3701501
Median	8288873	7774164	7080634	6361998	4091629	5363690	4645668	4920764
75% Percentile	8920686	10832444	8185544	6599518	6460573	5648267	5377488	5626840

IL-6 qPCR

	LSCs				AN-LSCs			
Travoprost	Control	0.078μg/mL	0.156μg/mL	0.313μg/mL	Control	0.078μg/mL	0.156μg/mL	0.313μg/mL
Donor 1	0.17	0.22	0.18	0.2	2.35	2.52	2.52	2.79
Donor 2	1.59	1.27	1.57	1.89	1.08	1	1.26	1.14
Donor 3	3.43	2.58	2.96	3.13	3.14	3.75	1.99	1.76
Donor 4	0.59	0.82	0.82	0.54	1.89	1.94	1.86	1.38
Donor 5	0.35	0.45	0.49	0.35	1.02	1.51	1.31	0.49
Donor 6	5.06	4.5	3.57	3.06	4.85	5.49	5	4.2
Donor 7					1.17	1.4	1.39	1.02
25% Percentile	0.305	0.3925	0.4125	0.3125	1.08	1.4	1.31	1.02
Median	1.09	1.045	1.195	1.215	1.89	1.94	1.86	1.38
75% Percentile	3.838	3.06	3.113	3.078	3.14	3.75	2.52	2.79

IL-6 ELISA

	LSCs				AN-LSCs			
Travoprost	Control	0.078μg/mL	0.156μg/mL	0.313μg/mL	Control	0.078μg/mL	0.156μg/mL	0.313μg/mL
Donor 1	217.745	206.325	213.775	189.885	388.52	389.54	305.665	352.41
Donor 2	57.281	55.721	56.581	56.106	267.425	283.415	288.115	378.77
Donor 3	189.27	173.05	180.21	174.63	213.27	198.89	210.31	212.17
Donor 4	166.95	159.415	157.71	146.085	355.64	375.13	390.305	372.565
Donor 5	265.775	269.75	269.44	262.06	102.5385	104.385	100.4465	99.346
Donor 6	434.89	501.06	472.64	449.11	385.46	342.21	311.03	329.79
Donor 7	412.27	433.46	393.93	367.06	351.85	387.03	304.46	305.69
25% Percentile	167	159.4	157.7	146.1	213.3	198.9	210.3	212.2
Median	217.7	206.3	213.8	189.9	351.9	342.2	304.5	329.8
75% Percentile	412.3	433.5	393.9	367.1	385.5	387	311	372.6

IL-8 qPCR

	LSCs				AN-LSCs			
Travoprost	Control	0.078μg/mL	0.156μg/mL	0.313μg/mL	Control	0.078μg/mL	0.156μg/mL	0.313μg/mL
Donor 1	1.84	2.88	3.06	2.84	3.74	4.26	3.25	3.04
Donor 2	1.46	1.6	2.09	1.71	3	2.71	2.91	3.08
Donor 3	2.91	3.47	3.56	2.43	0.69	0.8	0.75	0.62
Donor 4	0.05	0.05	0.06	0.07	6.5	6.68	8.38	9.83
Donor 5	1.91	2.59	1.69	1.96	0.04	0.07	0.04	0.04
Donor 6	1.32	1.09	0.84	0.92	1.6	1.63	1.4	1.73
Donor 7					2.74	2.72	2.68	2.35
25% Percentile	1.003	0.83	0.645	0.7075	0.69	0.8	0.75	0.62
Median	1.65	2.095	1.89	1.835	2.74	2.71	2.68	2.35
75% Percentile	2.16	3.028	3.185	2.533	3.74	4.26	3.25	3.08

IL-8 ELISA

	LSCs				AN-LSCs			
Travoprost	Control	0.078μg/mL	0.156μg/mL	0.313μg/mL	Control	0.078μg/mL	0.156μg/mL	0.313μg/mL
Donor 1	350.6	305.545	338.77	367.095	978.295	806.02	735.165	785.065
Donor 2	893.42	824.92	710.21	792.205	984.46	864.61	774.565	700.23
Donor 3	793.58	840.2	887.605	855.3	1112.38	830.94	727.36	744.67
Donor 4	712.755	897.425	846.9	980.85	274.89	237.915	196.98	189.705
Donor 5	729.205	1008.265	820.585	915.87	1329.74	881.975	943.695	887.265
Donor 6	542.17	523.305	567.6	561.99	579	537.035	496.86	499.935
Donor 7	306.3	319.99	232.335	253.87	561.09	535.98	495.13	492.32
25% Percentile	350.6	320	338.8	367.1	561.1	536	495.1	492.3
Median	712.8	824.9	710.2	792.2	978.3	806	727.4	700.2
75% Percentile	793.6	897.4	846.9	915.9	1112	864.6	774.6	785.1

TNF-α qPCR

	LSCs				AN-LSCs			
Travoprost	Control	0.078μg/mL	0.156μg/mL	0.313μg/mL	Control	0.078μg/mL	0.156μg/mL	0.313μg/mL
Donor 1	0.27	0.75	0.26	0.51	2.06	2.92	2.13	1.42
Donor 2	0.44	0.83	1.83	1.06	0.83	0.39	0.21	0.84
Donor 3	1.27	1.41	1.32	1.04	1.54	1.22	1.77	0.86
Donor 4								
Donor 5								
Donor 6	6.54	2.58	8.63	7.18	0.07	0.34	0.74	1.15
Donor 7					4.42	3.42	2.19	3.65
25% Percentile	0.3125	0.77	0.525	0.6425	0.45	0.365	0.475	0.85
Median	0.855	1.12	1.575	1.05	1.54	1.22	1.77	1.15
75% Percentile	5.223	2.288	6.93	5.65	3.24	3.17	2.16	2.535

PTGES2 qPCR

	LSCs				AN-LSCs			
Travoprost	Control	0.078μg/mL	0.156μg/mL	0.313μg/mL	Control	0.078μg/mL	0.156μg/mL	0.313μg/mL
Donor 1	1.9	1.64	1.27	1.53	1.2	0.87	1.17	1.18
Donor 2	0.6	0.92	0.85	0.92	0.71	0.89	0.96	0.81
Donor 3	0.96	0.53	0.81	0.7	1.03	1.67	1.75	1.81
Donor 4	1.6	0.91	1.41	0.92	2.48	1.11	1.29	1.87
Donor 5	2.9	3.72	3.35	3.5	1.14	1.06	0.62	0.73
Donor 6	1.53	0.69	1.39	2.09	0.95	1.35	1.2	1.02
Donor 7	0.13	1	0.92	0.84	0.8	1.17	0.62	1.14
25% Percentile	0.6	0.69	0.85	0.84	0.8	0.89	0.62	0.81
Median	1.53	0.92	1.27	0.92	1.03	1.11	1.17	1.14
75% Percentile	1.9	1.64	1.41	2.09	1.2	1.35	1.29	1.81

PTGES2 Western blot

	LSCs				AN-LSCs			
Travoprost	Control	0.078μg/mL	0.156μg/mL	0.313μg/mL	Control	0.078μg/mL	0.156μg/mL	0.313μg/mL
Donor 1	2218589	2956733.58	2408644.3	2519854.14	2730985.73	4913791.32	5163484.62	8252382.04
Donor 2	9812495	10634811.4	10318627.9	7109741.48	7662276.65	6450772.34	4377369.19	4729126.8
Donor 3	14800087	13159583.1	15574067.7	15967002.6	12522964.2	12685748	13260964.3	12571931.3
Donor 4	8728332	7868759.48	10078150.7	5072864.54	3364257.63	3327392.56	3339859.26	5900438.99
Donor 5	5037841	6216592.34	5331435.19	3868022.65	5274397.52	5738950.8	4487138.24	4753304.16
Donor 6	5491431	4840377.09	6176430.83	5634335.91	4920016.73	4810446.1	5421535.35	6679286.86
Donor 7	6104783	6356677.06	6508669.71	6729497.67	8668472.13	9112632.6	5739844.95	6796488.31
25% Percentile	5037841	4840377	5331435	3868023	3364258	4810446	4377369	4753304
Median	6104783	6356677	6508670	5634336	5274398	5738951	5163485	6679287
75% Percentile	9812495	10634811	10318628	7109741	8668472	9112633	5739845	8252382

PTGFR qPCR

	LSCs				AN-LSCs			
Travoprost	Control	0.078μg/mL	0.156μg/mL	0.313μg/mL	Control	0.078μg/mL	0.156μg/mL	0.313μg/mL
Donor 1	0.89	2.43	1.75	2.57	0.57	1.95	2.38	1.67
Donor 2	1.16	2.89	3.93	4.3	0.99	1.41	0.96	3.32
Donor 3	0.35	0.9	0.87	1.37	0.81	6.98	6.47	4.57
Donor 4	1.23	6.07	9.21	5.34	6.22	6.17	6.58	4.24
Donor 5	0.66	1.28	2.76	1.08	1.53	6.62	4.94	4.09
Donor 6	3.99	5.52	9.25	10.92	0.97	3.95	3.51	2.48
Donor 7	0.88	3.15	3.48	3.77	0.91	4.47	3.21	8.27
25% Percentile	0.66	1.28	1.75	1.37	0.81	1.95	2.38	2.48
Median	0.89	2.89	3.48	3.77	0.97	4.47	3.51	4.09
75% Percentile	1.23	5.52	9.21	5.34	1.53	6.62	6.47	4.57

6. Mitogen-activated protein kinases (MAPKs)

ERK2(MAPK1) qPCR

	LSCs				AN-LSCs			
Travoprost	Control	0.078μg/mL	0.156μg/mL	0.313μg/mL	Control	0.078μg/mL	0.156μg/mL	0.313μg/mL
Donor 1	1.87	1.19	1.17	1.62	1.2	1.32	1.26	1.48
Donor 2	0.29	0.56	0.59	0.73	0.74	1.12	0.92	0.85
Donor 3	1.26	1.29	1.04	1.07	0.88	1.03	1.19	1.26
Donor 4	2.1	1.17	1.75	1.17	1.67	1.18	1.25	2.06
Donor 5	1.95	1.92	2.74	2.14	3.98	2.57	2.57	2.46
Donor 6	1.25	0.47	1.11	1.32	1.23	1.33	1.58	1.12
Donor 7	0.28	1.05	1.25	0.9	0.63	0.94	0.51	0.93
25% Percentile	0.29	0.56	1.04	0.9	0.74	1.03	0.92	0.93
Median	1.26	1.17	1.17	1.17	1.2	1.18	1.25	1.26
75% Percentile	1.95	1.29	1.75	1.62	1.67	1.33	1.58	2.06

ERK1(MAPK3) qPCR

Travoprost	Control	0.078μg/mL	0.156μg/mL	0.313μg/mL	Control	0.078μg/mL	0.156μg/mL	0.313μg/mL
Donor 1	1.52	1.2	1.02	1.01	0.89	0.95	1	1.21
Donor 2	1.27	1.05	1.38	1.3	1.21	0.77	0.8	0.86
Donor 3	2.48	1.91	1.29	1.08	1.03	1.42	1.8	1.53
Donor 4	1.37	0.99	1.08	0.58	5.02	1.41	1.42	2.37
Donor 5	1.53	1.51	1.69	1.48	1.58	0.89	0.91	1.05
Donor 6	1.28	0.35	1.33	1.62	1.94	1.63	1.58	0.68
Donor 7	0.08	1.15	1.25	1.24	1.29	1.24	0.27	1.58
25% Percentile	1.27	0.99	1.08	1.01	1.03	0.89	0.8	0.86
Median	1.37	1.15	1.29	1.24	1.29	1.24	1	1.21
75% Percentile	1.53	1.51	1.38	1.48	1.94	1.42	1.58	1.58

JNK(MAPK8) qPCR

	LSCs				AN-LSCs			
Travoprost	Control	0.078μg/mL	0.156μg/mL	0.313μg/mL	Control	0.078μg/mL	0.156μg/mL	0.313μg/mL
Donor 1	1.34	1.33	1.23	0.94	0.39	0.69	0.84	1.11
Donor 2	0.59	0.61	0.66	0.42	0.31	0.27	0.44	0.57
Donor 3	0.76	0.58	0.63	0.49	0.45	0.58	1.01	1.06
Donor 4	1.1	0.76	0.99	0.73	0.4	0.56	0.57	1.21
Donor 5	1.82	1.76	2.16	1.58	0.66	1.28	1.76	2.4
Donor 6	1.22	1.23	1.33	1.24	0.39	0.48	0.65	1.36
Donor 7	0.67	1	0.83	0.88	0.4	0.89	1.04	1.05
25% Percentile	0.67	0.61	0.66	0.49	0.39	0.48	0.57	1.05
Median	1.1	1	0.99	0.88	0.4	0.58	0.84	1.11
75% Percentile	1.34	1.33	1.33	1.24	0.45	0.89	1.04	1.36

JNK1/2 Western blot

	LSCs				AN-LSCs			
Travoprost	Control	0.078μg/mL	0.156μg/mL	0.313μg/mL	Control	0.078μg/mL	0.156μg/mL	0.313μg/mL
Donor 1	26180356	33473273.9	38438267.1	37214302.2	31171384.1	35433059.8	36431371.5	36337040.7
Donor 2	36233780	36517965.5	34899728.5	33278386.8	37621346.4	33796350.1	36531277.5	37855760.8
Donor 3	32724630	27008226	29974353.1	28067489	29644164.2	31294829.3	27788012.9	35091047.3
Donor 4	22171319	21031290.2	27679262.1	20722286.9	32429761.9	27347910.5	30914171.9	30068339.6
Donor 5	24724908	28531468.6	30305164.4	30713716.3	27134383.8	35666583.2	29127235.3	34364792
Donor 6	9499064	10212011.1	12147896.2	11596734.8	17175971	16099146.2	18008419.2	20178039.1
Donor 7	15793114	20378051.2	20680804.6	23049975.5	27335625.7	28817923.4	27683290.2	26903457.6
25% Percentile	15793114	20378051	20680805	20722287	27134384	27347911	27683290	26903458
Median	24724908	27008226	29974353	28067489	29644164	31294829	29127235	34364792
75% Percentile	32724630	33473274	34899729	33278387	32429762	35433060	36431372	36337041

p38(MAPK14) qPCR

	LSCs				AN-LSCs			
Travoprost	Control	0.078μg/mL	0.156μg/mL	0.313μg/mL	Control	0.078μg/mL	0.156μg/mL	0.313μg/mL
Donor 1	1.3	0.91	0.83	0.93	0.63	0.75	0.88	1.08
Donor 2	0.9	0.82	1	1.09	0.74	0.66	0.83	0.56
Donor 3	2.21	0.93	1.08	1.31	0.6	0.99	1.1	1.2
Donor 4	1.17	0.56	1.17	0.59	3.06	1.03	1.1	1.92
Donor 5	1.66	1.37	1.87	1.51	1.58	1	1.15	0.99
Donor 6	0.99	0.42	1.1	1.19	0.98	1.02	1.07	0.77
Donor 7	0.2	1.04	0.8	1	1.2	1.59	0.83	1.43
25% Percentile	0.9	0.56	0.83	0.93	0.63	0.75	0.83	0.77
Median	1.17	0.91	1.08	1.09	0.98	1	1.07	1.08
75% Percentile	1.66	1.04	1.17	1.31	1.58	1.03	1.1	1.43

7. Matrix metalloproteinases (MMPs)

MMP-2 qPCR

	LSCs				AN-LSCs			
Travoprost	Control	0.078μg/mL	0.156μg/mL	0.313μg/mL	Control	0.078μg/mL	0.156μg/mL	0.313μg/mL
Donor 1	0.96	0.81	0.61	0.63	1.46	1.09	1.28	1.36
Donor 2	1.05	0.9	1.05	1.03	0.38	0.22	0.49	0.24
Donor 3	3.86	1.86	1.56	1.58	0.69	0.91	1.43	1.46
Donor 4	2.92	2.52	2.21	0.5	2.13	0.55	0.77	0.95
Donor 5	1.58	1.67	1.55	2.18	1.2	0.61	0.68	0.89
Donor 6	2.02	0.13	1.68	1.83	1.82	0.78	1.11	0.33
Donor 7	0.03	0.53	0.62	0.61	1.56	1.18	0.22	2.01
25% Percentile	0.96	0.53	0.62	0.61	0.69	0.55	0.49	0.33
Median	1.58	0.9	1.55	1.03	1.46	0.78	0.77	0.95
75% Percentile	2.92	1.86	1.68	1.83	1.82	1.09	1.28	1.46

MMP-2 ELISA

	LSCs				AN-LSCs			
Travoprost	Control	0.078μg/mL	0.156μg/mL	0.313μg/mL	Control	0.078μg/mL	0.156μg/mL	0.313μg/mL
Donor 1	33.15	32.92	29.94	32.83	15.02	14.7	14.99	15.25
Donor 2	20.48	23.02	23.53	23.4	23.98	22.87	24.09	25.54
Donor 3	30.16	30.07	29.83	30.83	32.38	31.81	31.47	30.54
Donor 4	31.03	34.53	31.27	30.69	23.65	21.84	22.03	22.62
Donor 5	22.72	24.22	24.69	24.64	30.99	31.94	29.66	32.63
Donor 6	11.59	11.48	11.57	9.7	27.17	25.98	28.29	30.66
Donor 7	28.67	29.17	30.98	31.27	19.59	17.18	18.83	20.3
25% Percentile	20.48	23.02	23.53	23.4	19.59	17.18	18.83	20.3
Median	28.67	29.17	29.83	30.69	23.98	22.87	24.09	25.54
75% Percentile	31.03	32.92	30.98	31.27	30.99	31.81	29.66	30.66

MMP-3 qPCR

	LSCs				AN-LSCs			
Travoprost	Control	0.078μg/mL	0.156μg/mL	0.313μg/mL	Control	0.078μg/mL	0.156μg/mL	0.313μg/mL
Donor 1	8.84	10.67	11.8	9.32	13.24	10.68	13.76	12.13
Donor 2	0.31	0.29	0.29	0.21	12.7	11.26	22.24	13.24
Donor 3	1.65	1.71	1.47	1.82	0.7	0.78	0.85	0.77
Donor 4	0.3	0.35	0.31	0.33	3.08	4.35	3.95	4.05
Donor 5	1.24	1.19	1.41	1.21	0.04	0.06	0.04	0.06
Donor 6	1.8	1.88	1.4	2.3	1.6	1.41	1.23	1.26
Donor 7	0.32	0.33	0.11	0.09	3.52	4.14	4.01	5.04
25% Percentile	0.31	0.33	0.29	0.21	0.7	0.78	0.85	0.77
Median	1.24	1.19	1.4	1.21	3.08	4.14	3.95	4.05
75% Percentile	1.8	1.88	1.47	2.3	12.7	10.68	13.76	12.13

MMP-3 ELISA

	LSCs				AN-LSCs			
Travoprost	Control	0.078μg/mL	0.156μg/mL	0.313μg/mL	Control	0.078μg/mL	0.156μg/mL	0.313μg/mL
Donor 1	299.81	265.25	276.39	398.35	1975.37	1865.75	1866.96	1681.13
Donor 2	2020.4	1986.17	1778.71	2208.88	1748.85	1290.23	1396.68	1659.39
Donor 3	630.54	625.01	569.98	648.2	1657.34	1517.49	1430.45	1334.26
Donor 4	1236.87	1343.59	872.63	1060.65	69.66	58.16	73.94	50.31
Donor 5	780.405	924.31	832.15	870.28	741.005	810.935	602.61	518.08
Donor 6	85.44	108.17	38.565	36.945	1870.84	1786.465	1820.11	1902.61
Donor 7	1406.58	1209.465	1340.495	1365.465	1870.7	1662.91	1728.925	1714.175
25% Percentile	299.8	265.3	276.4	398.4	741	810.9	602.6	518.1
Median	780.4	924.3	832.2	870.3	1749	1517	1430	1659
75% Percentile	1407	1344	1340	1365	1871	1786	1820	1714

MMP-9 qPCR

	LSCs				AN-LSCs			
Travoprost	Control	0.078μg/mL	0.156μg/mL	0.313μg/mL	Control	0.078μg/mL	0.156μg/mL	0.313μg/mL
Donor 1	3.22	3.73	4.26	4.13	15.96	16.64	16.45	18.27
Donor 2	1.2	2.61	1.84	0.79	5.22	5.7	4.17	3.76
Donor 3	0.12	0.55	0.95	0.53	5.24	7.32	6.72	8.08
Donor 4	2.55	1.85	1.03	0.71	1.89	2.1	1.7	1.66
Donor 5								
Donor 6	0.92	3.61	2.5	2.34	1.04	1.36	2.01	2.8
Donor 7					1.43	1.04	1.24	0.8
25% Percentile	0.52	1.2	0.99	0.62	1.333	1.28	1.585	1.445
Median	1.2	2.61	1.84	0.79	3.555	3.9	3.09	3.28
75% Percentile	2.885	3.67	3.38	3.235	7.92	9.65	9.153	10.63

MMP-9 ELISA

	LSCs				AN-LSCs			
Travoprost	Control	0.078μg/mL	0.156μg/mL	0.313μg/mL	Control	0.078μg/mL	0.156μg/mL	0.313μg/mL
Donor 1	142.975	136.35	135.145	133.1	170.37	134.545	153.235	155.77
Donor 2	149.85	164.36	147.075	140.205	148.04	160.49	158.305	155.425
Donor 3	127.825	130.575	129.525	111.735	119.53	137.44	158.065	179.43
Donor 4	126.86	129.25	108.38	120.37	111.255	124.085	122.79	211.41
Donor 5	112.1075	113.425	110.29	112.1075	96.09	117.87	134.425	133.27
Donor 6	140.415	126.63	137.505	169.155	135.105	136.185	124.26	133.53
Donor 7	135.105	158.835	130.08	125.31	147.885	129.285	115.005	159.03
25% Percentile	126.9	126.6	110.3	112.1	111.3	124.1	122.8	133.5
Median	135.1	130.6	130.1	125.3	135.1	134.5	134.4	155.8
75% Percentile	143	158.8	137.5	140.2	148	137.4	158.1	179.4

8. Apoptosis-related genes

caspase-3 qPCR

	LSCs				AN-LSCs			
Travoprost	Control	0.078μg/mL	0.156μg/mL	0.313μg/mL	Control	0.078μg/mL	0.156μg/mL	0.313μg/mL
Donor 1	1.11	1.13	1.31	1.43	1.05	1.19	2.3	1.18
Donor 2	1.11	1.11	1.27	1.26	1.24	1.38	1.34	1.33
Donor 3	0.83	0.78	0.67	0.7	1.22	1.44	1.51	2.71
Donor 4	0.67	0.62	0.81	0.44	1.02	0.88	1.07	0.88
Donor 5	0.9	1.22	0.9	0.96	1.63	0.58	0.64	0.59
Donor 6	1.63	1.59	1.48	1.65	0.88	1.05	1.2	1.25
Donor 7					1.04	1.07	0.83	0.98
25% Percentile	0.79	0.74	0.775	0.635	1.02	0.88	0.83	0.88
Median	1.005	1.12	1.085	1.11	1.05	1.07	1.2	1.18
75% Percentile	1.24	1.313	1.353	1.485	1.24	1.38	1.51	1.33

caspase-3 Western blot

	LSCs				AN-LSCs			
Travoprost	Control	0.078μg/mL	0.156μg/mL	0.313μg/mL	Control	0.078μg/mL	0.156μg/mL	0.313μg/mL
Donor 1	3445476	1989907.98	2057979.98	2449016.03	1682679.58	853839.526	1576355.08	1345258.9
Donor 2	806042	834746.398	952983.146	763329.614	482146.71	577067.568	529152.296	437217.342
Donor 3	3233996	3008090.36	1964765.31	1602415.8	1836382.84	2038195.71	2525815.99	2010617.94
Donor 4	4253777	4105559.78	4405514.06	4264517.77	7794969.98	5860193.36	6437853.01	7353918.62
Donor 5	941594	995116.211	809257.024	1083349.94	1175902.15	1266730.64	1159803.1	1191399.06
Donor 6	2105137	801739.64	862880.816	915619.308	1231127.86	743411.268	760813.076	2030299.76
Donor 7	5952078	7198003.88	5417733.48	3551054.6	3605156.36	3365386.76	3565318.85	3307074.29
25% Percentile	941594	834746	862881	915619	1175902	743411	760813	1191399
Median	3233996	1989908	1964765	1602416	1682680	1266731	1576355	2010618
75% Percentile	4253777	4105560	4405514	3551055	3605156	3365387	3565319	3307074

Bcl-2 qPCR

	LSCs				AN-LSCs			
Travoprost	Control	0.078μg/mL	0.156μg/mL	0.313μg/mL	Control	0.078μg/mL	0.156μg/mL	0.313μg/mL
Donor 1	0.35	0.69	0.6	0.43	1.2	2.2	1.67	1.89
Donor 2	0.47	0.45	0.55	0.66	0.89	0.97	0.77	0.51
Donor 3	1.55	0.99	1.01	1.02	0.7	0.58	0.97	1.3
Donor 4	4.79	5.64	5.36	1.02	0.57	0.55	0.63	0.6
Donor 5	0.46	1.02	1.1	0.59	4.26	1.47	0.97	1.45
Donor 6	1.81	1.46	1.54	1.54	0.72	1.37	1.13	1.05
Donor 7					1.23	1.34	0.72	1.2
25% Percentile	0.4325	0.63	0.5875	0.55	0.7	0.58	0.72	0.6
Median	1.01	1.005	1.055	0.84	0.89	1.34	0.97	1.2
75% Percentile	2.555	2.505	2.495	1.15	1.23	1.47	1.13	1.45

Bax qPCR

	LSCs				AN-LSCs			
Travoprost	Control	0.078μg/mL	0.156μg/mL	0.313μg/mL	Control	0.078μg/mL	0.156μg/mL	0.313μg/mL
Donor 1	2.16	2.13	2.49	2.4	1.17	1.9	1.5	1.51
Donor 2	1.39	1.44	0.74	0.74	1.6	1.79	1.35	2.07
Donor 3	1.15	1.17	1.02	1.06	1.88	1.82	3.91	3.16
Donor 4	0.92	0.93	0.67	0.11	1.72	0.86	0.78	1.01
Donor 5	0.28	0.51	0.56	0.46	0.67	0.41	0.41	0.46
Donor 6	1.11	0.42	1.65	1.6	0.93	1.51	1	0.66
Donor 7					1.12	1.04	0.29	1.05
25% Percentile	0.76	0.4875	0.6425	0.3725	0.93	0.86	0.41	0.66
Median	1.13	1.05	0.88	0.9	1.17	1.51	1	1.05
75% Percentile	1.583	1.613	1.86	1.8	1.72	1.82	1.5	2.07

9. Retinoic acid signaling pathway-related genes

ADH-7 qPCR

	LSCs				AN-LSCs			
Travoprost	Control	0.078μg/mL	0.156μg/mL	0.313μg/mL	Control	0.078μg/mL	0.156μg/mL	0.313μg/mL
Donor 1	2.32	2.13	2.06	2.35	1.56	1.27	1.15	1.46
Donor 2	0.55	0.51	0.46	0.57	1.07	1.62	1.02	1.44
Donor 3	2.38	2.79	2.32	2.07	3.61	4.6	4.95	4.08
Donor 4	0.09	0.12	0.11	0.14	9.25	8.57	9.97	13.16
Donor 5	1.44	1.75	1.88	1.34				
Donor 6	2.54	2.24	2.63	3.24	10.72	8.08	9.93	10.07
Donor 7					4.26	4.14	4.32	5
25% Percentile	0.435	0.4125	0.3725	0.4625	1.438	1.533	1.118	1.455
Median	1.88	1.94	1.97	1.705	3.935	4.37	4.635	4.54
75% Percentile	2.42	2.378	2.398	2.573	9.618	8.203	9.94	10.84

ADH-7 Western blot

	LSCs				AN-LSCs			
Travoprost	Control	0.078μg/mL	0.156μg/mL	0.313μg/mL	Control	0.078μg/mL	0.156μg/mL	0.313μg/mL
Donor 1	7485366	3965929.89	5207450.83	3724395.12	3639735.21	3396926.23	5721254.15	7324903.44
Donor 2	16577823	12344830	11352998.1	7892012.02	9329781.01	9828127.25	9260796.19	11364751.1
Donor 3	13980551	8967438.76	9781297.96	9261196.18	8014170.68	11433270.3	8125001.86	8466654.49
Donor 4	14381256	11389143	12188903.3	11047002.4	12356281.8	11252347.8	12059160.9	14783297.7
Donor 5	16417810	15266604.5	16383471.4	12888326.7	10301532.2	10005362.5	8560118.14	16816091.5
Donor 6	15283938	9564681.98	8245938.78	8775079.23	7237989.65	5892196.24	7151897.74	9121109.51
Donor 7	16906750	15248508.7	15239416	10006195.9	6300621.91	8281123.69	10375381.7	12004336.3
25% Percentile	13980551	8967439	8245939	7892012	6300622	5892196	7151898	8466654
Median	15283938	11389143	11352998	9261196	8014171	9828127	8560118	11364751
75% Percentile	16577823	15248509	15239416	11047002	10301532	11252348	10375382	14783298

FABP5 qPCR

	LSCs				AN-LSCs			
Travoprost	Control	0.078μg/mL	0.156μg/mL	0.313μg/mL	Control	0.078μg/mL	0.156μg/mL	0.313μg/mL
Donor 1	2.62	2.51	2.88	3.52	3.4	2.76	3.2	4.86
Donor 2	1.24	0.99	1	1.4	4.98	5.96	3.97	6.58
Donor 3	0.96	1.05	0.94	1.35	1.99	1.84	2.2	3.57
Donor 4	0.18	0.16	0.18	0.24	1.38	1.97	2.33	2.78
Donor 5	1.21	1.31	1.27	1.19	1.34	0.6	0.83	0.95
Donor 6	1.5	1.48	1.34	1.57	1.18	0.95	0.67	0.94
Donor 7					2.41	2.59	2.21	1.91
25% Percentile	0.765	0.7825	0.75	0.9525	1.34	0.95	0.83	0.95
Median	1.225	1.18	1.135	1.375	1.99	1.97	2.21	2.78
75% Percentile	1.78	1.738	1.725	2.058	3.4	2.76	3.2	4.86

FABP5 Western blot

	LSCs				AN-LSCs			
Travoprost	Control	0.078μg/mL	0.156μg/mL	0.313μg/mL	Control	0.078μg/mL	0.156μg/mL	0.313μg/mL
Donor 1	4020001	4034255.92	2476640.56	2034738.68	2222405.63	1441275.18	1645824	1654605.31
Donor 2	4605824	6630869.36	7677015.92	6715364.81	3851211.43	4227985.36	6129648.38	3485962.84
Donor 3	369118	472701.807	426655.102	709585.938	1265923.82	1231254.43	1580917.29	2163741.69
Donor 4	2716265	2717860.34	2534037.12	2422507.11	5230312.93	4427011.44	4789231.48	6479810.48
Donor 5	6571405	7441962.89	6361421.44	6870025.47	5589000	6123158.25	4957289.98	5278143.19
Donor 6	3669812	3258569.37	3677379.59	3128873.41	5508746.49	3541825.35	5031090.53	5890290.97
Donor 7	984119	1314717.48	949165.764	689650.964	1710712.9	1928856.27	2317971.35	1773324.54
25% Percentile	984119	1314717	949166	709586	1710713	1441275	1645824	1773325
Median	3669812	3258569	2534037	2422507	3851211	3541825	4789231	3485963
75% Percentile	4605824	6630869	6361421	6715365	5508746	4427011	5031091	5890291

ALDH1A1 qPCR

	LSCs				AN-LSCs			
Travoprost	Control	0.078μg/mL	0.156μg/mL	0.313μg/mL	Control	0.078μg/mL	0.156μg/mL	0.313μg/mL
Donor 1	0.73	0.83	0.73	0.91	0.48	0.44	0.46	0.62
Donor 2	0.81	0.85	0.81	0.83	2.54	2.52	2.73	2.99
Donor 3	6.8	8.35	8.31	6.93	0.5	0.6	0.69	0.74
Donor 4	0.23	0.18	0.19	0.27	6.98	7.63	6.52	7.01
Donor 5	2.43	2.81	2.51	2.76	0.2	0.14	0.16	0.17
Donor 6	0.44	0.54	0.38	0.46	0.6	0.58	0.59	0.61
Donor 7					3.79	4.44	3.86	3.98
25% Percentile	0.3875	0.45	0.3325	0.4125	0.48	0.44	0.46	0.61
Median	0.77	0.84	0.77	0.87	0.6	0.6	0.69	0.74
75% Percentile	3.523	4.195	3.96	3.803	3.79	4.44	3.86	3.98

CRABP-2 qPCR

	LSCs				AN-LSCs			
Travoprost	Control	0.078μg/mL	0.156μg/mL	0.313μg/mL	Control	0.078μg/mL	0.156μg/mL	0.313μg/mL
Donor 1	1.8	1.43	1.36	1.68	1.37	1.14	1.17	1.39
Donor 2	0.03	0.02	0.02	0.02	1.78	1.7	1.59	1.68
Donor 3	1.94	1.47	1.62	1.35	1.58	1.73	1.85	2.12
Donor 4	1.84	1.71	1.64	1.25	4.57	2.51	3.34	4.43
Donor 5	1.4	1.41	1.48	1.19	2.87	2.49	2.61	3.18
Donor 6	2.56	1.19	2.4	2.61	2.45	2.92	2.93	2.17
Donor 7	1.3	6.09	3.5	2.28	0.03	0.03	0.05	0.03
25% Percentile	1.3	1.19	1.36	1.19	1.37	1.14	1.17	1.39
Median	1.8	1.43	1.62	1.35	1.78	1.73	1.85	2.12
75% Percentile	1.94	1.71	2.4	2.28	2.87	2.51	2.93	3.18

CRABP-2 Western blot

	LSCs				AN-LSCs			
Travoprost	Control	0.078μg/mL	0.156μg/mL	0.313μg/mL	Control	0.078μg/mL	0.156μg/mL	0.313μg/mL
Donor 1	5012364	6770744.73	5917707.59	4909092.18	5622832.34	5908376.32	6251591.64	6839253.89
Donor 2	4236422	2424398.91	3032925.14	2051289.58	2296473.35	3343451.65	3792436.43	3994489.91
Donor 3	5652492	4418228.07	5060442.71	4186350.4	5195485.57	5020106.59	6889749.26	9806172.3
Donor 4	4569338	6408769.93	5429884.67	4320021.25	4365808.3	6161932.51	6805982.22	4781573.58
Donor 5	3508833	2388731.83	2479657.53	2718046.48	4300989	3371762.66	4215894.12	3672035.87
Donor 6	6260124	6343928.81	4814982.21	5631493.92	4404798.42	4989870.86	5999408.08	6139939.82
Donor 7	7350158	6561809.58	6430698.29	7234230.77	7442974.97	6820313.87	8091317.21	7584852.4
25% Percentile	4236422	2424399	3032925	2718046	4300989	3371763	4215894	3994490
Median	5012364	6343929	5060443	4320021	4404798	5020107	6251592	6139940
75% Percentile	6260124	6561810	5917708	5631494	5622832	6161933	6889749	7584852

PPARγ qPCR

	LSCs				AN-LSCs			
Travoprost	Control	0.078μg/mL	0.156μg/mL	0.313μg/mL	Control	0.078μg/mL	0.156μg/mL	0.313μg/mL
Donor 1	1.04	0.95	1	1.03	0.06	0.06	0.07	0.07
Donor 2	0.67	0.56	0.65	0.47	0.79	0.49	0.6	0.97
Donor 3	0.37	0.38	0.32	0.4	2.07	1.93	2.17	1.96
Donor 4	0.37	0.43	0.38	0.48	1.62	1.95	1.81	2.27
Donor 5	6.19	6.18	8.65	5.37	0.5	0.5	0.57	0.58
Donor 6	1.54	2.07	1.7	1.77	3.88	3.92	3.63	3.66
Donor 7	1.1	0.75	0.63	0.54	1.72	1.58	1.68	1.72
25% Percentile	0.37	0.43	0.38	0.47	0.5	0.49	0.57	0.58
Median	1.04	0.75	0.65	0.54	1.62	1.58	1.68	1.72
75% Percentile	1.54	2.07	1.7	1.77	2.07	1.95	2.17	2.27

PPARγ Western blot

	LSCs				AN-LSCs			
Travoprost	Control	0.078μg/mL	0.156μg/mL	0.313μg/mL	Control	0.078μg/mL	0.156μg/mL	0.313μg/mL
Donor 1	6172891	6530731.1	5610088.61	4936291.72	5728636.15	5041926.14	5050539.98	6369510.18
Donor 2	4868104	4072802.36	5330363.13	2512658.32	4206197.97	4280386.78	4168762.84	4430762.25
Donor 3	4418545	3242854.49	3484119.79	4539456.31	4355492.54	4545607.95	4242783.94	4924353.6
Donor 4	6622418	6391048.37	8398568.81	7453472.78	9831833.94	8416929.75	8309626.67	10560634
Donor 5	3245786	3094793.71	3772656.48	5636902.26	6412127.92	4241267.73	3601401.47	3451057.39
Donor 6	3826596	4288457.17	4455723.32	4363380.11	6328460.39	5638947.71	6240638.38	7449469.41
Donor 7	5573467	6217066.82	5846941.71	3550822.84	6037889.65	5959543.8	4167187.77	5107081.18
25% Percentile	3826596	3242854	3772656	3550823	4355493	4280387	4167188	4430762
Median	4868104	4288457	5330363	4539456	6037890	5041926	4242784	5107081
75% Percentile	6172891	6391048	5846942	5636902	6412128	5959544	6240638	7449469

VEGFA qPCR

	LSCs				AN-LSCs			
Travoprost	Control	0.078μg/mL	0.156μg/mL	0.313μg/mL	Control	0.078μg/mL	0.156μg/mL	0.313μg/mL
Donor 1	1.11	2.09	2.57	1.46	2.32	3.58	1.87	3.08
Donor 2	0.95	1.55	0.86	0.89	5.13	3.86	3.35	3.97
Donor 3	1.58	1.04	0.78	0.9	0.62	0.51	1.26	1.41
Donor 4	0.57	0.6	0.73	0.03	2.01	1.13	1.23	1.42
Donor 5	0.84	1.4	1.57	1.13	0.32	0.17	0.22	0.14
Donor 6	1.24	0.51	1.64	1.22	1.4	2.62	2	1.27
Donor 7					2.49	2.63	0.47	2.89
25% Percentile	0.7725	0.5775	0.7675	0.675	0.62	0.51	0.47	1.27
Median	1.03	1.22	1.215	1.015	2.01	2.62	1.26	1.42
75% Percentile	1.325	1.685	1.873	1.28	2.49	3.58	2	3.08

VEGFA ELISA

	LSCs				AN-LSCs			
Travoprost	Control	0.078μg/mL	0.156μg/mL	0.313μg/mL	Control	0.078μg/mL	0.156μg/mL	0.313μg/mL
Donor 1	1074.5	1210.35	1236.35	913.275	1252.35	1281.75	1171.75	1360.1
Donor 2	466.125	461.59	447.09	368.8	1161.7	892.93	977.245	1587
Donor 3	809.28	756.73	699.36	782.52	471.51	521.945	578.895	486.35
Donor 4	312.895	295.09	353.34	394.51	65.2315	70.1165	60.1155	74.3745
Donor 5	464	498	369	300	779.13	603	600.33	765.565
Donor 6					376	436	321	398.5
Donor 7					423.84	571.81	374.64	516.97
25% Percentile	388.4	378.3	361.2	334.4	376	436	321	398.5
Median	466.1	498	447.1	394.5	471.5	571.8	578.9	517
75% Percentile	941.9	983.5	967.9	847.9	1162	892.9	977.2	1360
