# Supplementary material for: Increased sensitivity of primary aniridia limbal stromal cells to travoprost, leading to elevated migration and MMP-9 protein levels, in vitro
Source: PLoS One. 2025 Jun 26;20(6):e0326967. doi: 10.1371/journal.pone.0326967 (PMC12200743; doi:10.1371/journal.pone.0326967)
Supplement: S2 File — (PDF) [file pone.0326967.s002.pdf]

DeepL

Subscribe to DeepL Pro to translate larger documents.
Visit www.DeepL.com/pro for more information.

Medical Association
of the Saarland

Corporate body
of public law

ÄrzteKammer dev Saarlandes Postfacfi 10 02 62- 66002 Saarbrücken Ethics Committee

Prof. Dr. med Nóra Szentmáry Dr. Rolf
M. Schwiete Center for
Limbal stem cell research and congenital aniridia
Saarland University Hospital
Kirrberger Str. 100, Building 22
66424 Homburg/Saar

Ethics Committee
Business pillars

Faktoreistrasse 4
66111 Saarbrücken

Processing:
D. Bunzeì - Phone (06 61) 40 03 - 218
N. Halder - Phone (06 81) 40 03 - 216

Teiefax (06 81) 40 03 - 43 94
E-Maii: efhikł'ommission@aeksaar.de
Internet: www.aerztekammer-saarland.de

Our Zeicńen:	Your letter dated:	Ear sign:	Date:
124/23			} j. i§lil ITU

Effect of antiglaucomatous eye drops on apoptosis, stem cell and differentiation markers **in human primary limbal epithelial cells and fibroblasts from** aniridia patients
Our identification no. 124/23 (Please quote in all correspondence!)

Dear Professor Szentmáry,

We refer to the letter sent to us by Ms. Hannah Rapp by e-mail on 14.06.2023in which the above-mentioned study is submitted to us for evaluation

The documents were examined by the Chairman in accordance with Section 8 of our Rules of Procedure; no deliberations were by the entire Commission.

There are no professional or ethical objections to conducting the above-mentioned study at the study center - **Dr. Rolf M. Schwiete Center for Limbal Stem Cell Research and Congenital Aniridia, Saarland University Hospital,** Kirrberger Str. 100, Building 22, 66424 **Homburg/Saar**.

We would like to draw your to the fact that the Ethics Committee's opinion is intended to assist you in assessing the ethical and legal aspects of a planned research project. The responsible medical director of the research project remains responsible the planning and implementation.

If changes are made to the research project before or during implementation, a corresponding application must be submitted again before the change is made. In the case of amendments, both the application number and the amended passages should be clearly marked in the relevant documents, as otherwise rapid processing is not possible. Newly participating trial centers must also reported to the Ethics Commission.

The Ethics Committee at the Saarland Medical Association operates in accordance with the international guidelines of the ICH, GCP, AMG and MDR, is recognized under state law (Saarland Medical Professions Act, Section 5 (1)) and is registered with the Federal Institute for Drugs and Medical Devices (Section 41a AMG) and the Federal Office for Radiation Protection in accordance with Section 36 (1) of the Radiation Protection Act.

Data protection aspects of research projects are generally only examined cursorily by the European Commission. This vote / this assessment therefore does not replace the consultation of the responsible company or government data protection .

Commerzbank Saarórücxen
IBAN: DE35 59D4 0000 0538 9200 00
LIC: COBADEFFXXX

apoBank Saarbrücken
IBAN: DE69 3006 0601 0001 9262 09
BIC: DAAEDEDOXXX

Bank 1 Saar Saarbrücken
IBAN: DE83 5919 0000 0001 5750 07
BIC: SABADESS

We expressly point out that the research project must be discontinued if there is a recognizable trend reversal with a negative result.

We wish them every success in their planned task and would be grateful if they could send us a final report in due course.

Yours sincerely

Prof. Dr. U. Grundmann Chairman

The valuation is based on the documents listed below:

- Clarification AN-Glaucoma 2023.docx
- Consent AN-Glaucoma 2023.docx
- EK-0002 Electronic checklist AN-Glaucoma 2023.doc
- E-mail cover letter dated 14.06.2023
